# Label-free and real-time monitoring of photoaging with high spatiotemporal resolution using an nIR fluorescent nanosensor array

**DOI:** 10.1126/sciadv.adt2296

**Published:** 2025-09-12

**Authors:** Youngwook Cho, Hwira Baek, Damee Koh, Changyu Tian, Minah Choi, Jung Woo, Junoh Kim, Seungchan Baek, Jin Woong Kim, Soo-Yeon Cho

**Affiliations:** ^1^ School of Chemical Engineering, Sungkyunkwan University (SKKU), Suwon 16419, Republic of Korea.; ^2^ Technology Innovation Center, Shinsegae International Inc., Seoul 06015, Republic of Korea.

## Abstract

Reactive oxygen species (ROS) bursts from photoaging cause skin damage and chronic conditions. Understanding spatiotemporal ROS dynamics is critical for developing therapies and cosmetic strategies to enhance skin health. Conventional assays and fluorescence microscopy lack the resolution for real-time ROS quantification due to photobleaching and labeling issues. Here, we developed a label-free, real-time monitoring platform with high spatiotemporal resolution using a near-infrared (nIR) fluorescent single-walled carbon nanotube (SWNT) nanosensor array to quantify ROS bursts from daily photoaging. The SWNT array, dual-functionalized with DNA and poly-l-lysine, achieved selective H_2_O_2_ recognition and skin cell compatibility. The skin cell–friendly nanosensor interface (SNI) enabled attomole-level detection of H_2_O_2_ bursts in a two-dimensional keratinocyte model under natural ultraviolet exposure, revealing photoadaptation behavior. Distinct oxidative stress wave profiles were identified via nIR data and numerical modeling. Using SNI, we introduced the anti-ROS score to evaluate skin care antioxidants, providing insights into photoaging pathways and cosmetic advancements.

## Teaser

A nIR nanosensor array allows label-free, real-time monitoring of ROS burst during photoaging with high spatiotemporal resolution.

## INTRODUCTION

Photoaging of the skin caused by solar ultraviolet (UV) exposure is a continuous and unavoidable process in our daily life, resulting in critical effects on skin health. It is primarily driven by UVA with wavelengths of 315 to 400 nm, allowing it to penetrate both the epidermis and dermis layers of the skin. UVA affects various types of underlying cells across the skin layers, with keratinocytes in the epidermis being the primary target, while fibroblasts, endothelial cells, and immune cells in the dermis are also affected (*1*, *2*). It triggers a cascade of cellular responses driven by immunomodulatory cytokines that alter the dermal microenvironment, leading to a burst in reactive oxygen species (ROS) production. Cellular chromophores such as porphyrins or heme-containing proteins (*3*), transfer an electron to molecular oxygen, producing oxygen free radicals, specifically superoxide (·O_2_^−^), which can subsequently lead to the formation of hydrogen peroxide (H_2_O_2_) via the type I photosensitization mechanism (*4*–*6*).

**Fig. 1.**
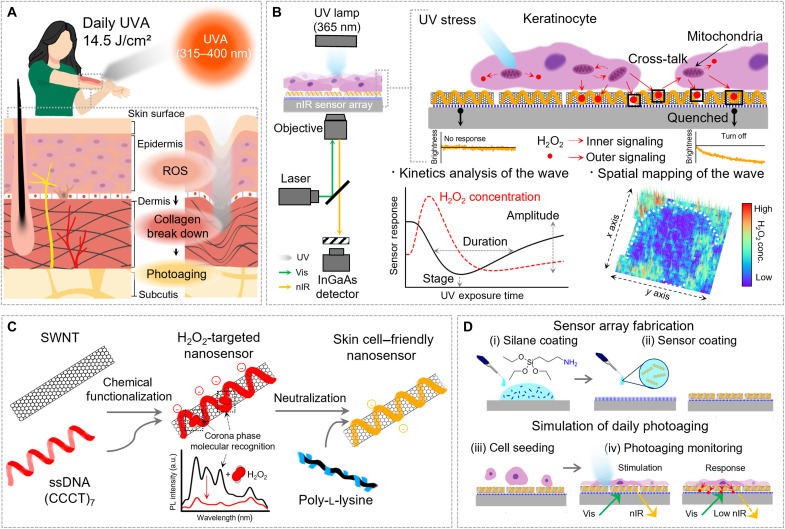
Schematic illustration of label-free and real-time spatiotemporal monitoring of photoaging using an nIR fluorescent SNI. (**A**) Skin photoaging mechanism induced by UVA radiation. (**B**) Instrumental setup for UVA radiation and real-time photoaging measurement of keratinocytes grown on a nanosensor interface (left). Intracellular stress response in single cells and extracellular ROS efflux in multi-cell systems under photoaging conditions (right, top). Kinetic and spatial analysis of skin photoaging through H_2_O_2_ efflux modeling and 3D mapping of the wave (right, bottom). (**C**) Synthesis flow of a cell-friendly label-free H_2_O_2_ nanosensor using SWNTs. (**D**) SNI fabrication and photoaging simulation methods.

This ROS burst up-regulates matrix metalloproteases (MMPs), which orchestrate the degradation of the extracellular matrix (*7*), resulting in abnormal collagen breakdown and disruption of the skin’s structural integrity (Fig. 1A) (*8*). This degradation damages the mature collagen, leading to a loss of skin elasticity, a hallmark of wrinkle formation associated with aging (*9*, *10*). ROS also contributes to pigmentation by oxidizing melanin precursors leading to increased melanin production and irregular skin tone (*11*). Furthermore, molecular changes such as DNA transversion driven by ROS burst can cause mutations in tumor suppressor genes such as p53 leading to chronic issues such as skin cancer (*12*–*14*). Hence, photoaging, distinct from intrinsic aging, induces premature skin deterioration driven by solar irradiation, frequently coexisting with age-related changes in skin appearance and function (*15*). Given its substantial risks, understanding the spatiotemporal dynamics of ROS production and their burst wave pattern in quantified way is essential for developing effective therapies and cosmetic strategies for photoaging that can be applied at the right time and location and in the correct amount to enhance skin health. However, fully understanding ROS kinetics in photoaging at high spatiotemporal resolution remains highly challenging because of the rapid chain reactions of short-lived ROS occurring on nano- to millisecond scale and their immediate response to environmental molecules (*16*, *17*). This makes it extremely difficult to accurately monitor the real-time spatiotemporal kinetics of ROS in the skin.

Conventional techniques for monitoring photoaging rely on fluorescence assay and microscopy with organic dyes such as 2,7-dichlorodihydrofluorescein diacetate (DCF-DA) to track changes in ROS levels in two-dimensional (2D) (*18*, *19*) or 3D skin models (*20*, *21*). While this method has been widely used for precise and reliable spatiotemporal tracking of ROS release, it has critical limitations in profiling the dynamic nature of photoaging under daily conditions. Although daily photoaging is driven by continuous low-power UV radiation [e.g., 7 to 13 J/cm^2^ over the course of a day (*22*)], many studies have performed static analyses with low temporal resolution with intervals of several minutes. This limitation arises from the photobleaching of dyes, which prevents stable and accurate signal calculations from live cells (*23*), making it difficult to precisely quantify the immediate generation of ROS waves on a millisecond timescale and their real-time communication between cells. In addition, the inherent dependence of fluorescent dye assays on endocytosis and cellular uptake leads to variability in the intracellular distribution and concentration of the dye, making accurate quantification of light intensity and ROS concentration unfeasible, while also limiting the ability to capture spatial dynamics of efflux waves due to the focus on intracellular information. Therefore, it requires the use of the UVB region with its higher energy (*24*–*26*) or longer time (*27*, *28*) [e.g., cumulatively repeated for 3 to 5 days (*29*)] and strong intensity of UVA exposure (*30*) to generate sufficient ROS levels across an unnecessarily broader spatial range and produce noticeable broad changes in the cellular environment. Moreover, labeling inherently introduces stress to skin cells, making it challenging to ascertain whether the observed effects are solely due to UV-induced stress. Therefore, there is a critical need for the development of analytical techniques capable of monitoring and accurately quantifying low-concentration ROS bursts in real-time under daily UV conditions, with millisecond-level resolution and in a label-free manner, while ensuring long-term signal stability without photobleaching.

In this study, we developed a label-free, real-time photoaging monitoring technique in high spatiotemporal resolution. We designed and fabricated a uniform, centimeter-scale, near-infrared (nIR) fluorescent single-walled carbon nanotubes (SWNTs)–based nanosensor array to detect ROS burst by photo-stress from a 2D skin model (e.g., keratinocyte layer) (Fig. 1B). This SWNTs nanosensor array exhibits instantaneous nIR response due to exciton quenching by the selective absorption of H_2_O_2_ in attomole level, which is the final product of ROS responsible for intra- and outer signaling in the skin cell’s stress response. The signal from this reaction can be quantified in real time as the local H_2_O_2_ concentration on the array through our numerical model, enabling the characterization of the quantified H_2_O_2_ efflux wave with various performance parameters under different UV stress conditions. Through 3D mapping, we can spatiotemporally observe the mechanisms of stress response and adaptation across skin cells, with a resolution of 500 ms temporally and 127 nm per square pixel spatially.

To synthesize a dual-function nanosensor that is simultaneously sensitive to H_2_O_2_ and biocompatible with skin cells, we functionalized SWNTs with single-stranded DNA (ssDNA) (CCCT)_7_ oligonucleotides (C-SWNTs) via π-π stacking interactions, forming a complex that creates specific corona sites for the selective molecular recognition of H_2_O_2_ (*31*, *32*), and then cofunctionalized them with poly-l-lysine (PLL) to ensure compatibility with skin cells (L-SWNTs) (Fig. 1C). As a cationic peptide, PLL electrostatically self-assembled with negatively charged C-SWNTs. When coated onto (3-aminopropyl) triethoxysilane (APTES)–treated glass substrates, the negatively charged parts of the L-SWNTs interact with the amine groups of the APTES layer, while the PLL-modified parts of the L-SWNTs align toward the cell-loading surface, providing an optimal condition for the negatively charged cell membranes to attach (*33*, *34*). This creates a skin cell–friendly nanosensor interface (SNI) for photoaging monitoring (Fig. 1D). The PLL- (CCCT)_7_ dual-functionalization corona construct of the SNI maintains high sensitivity and selectivity while enabling uniform and spatially continuous monitoring of nIR responses across the entire region of interest, offering enhanced capabilities compared to previous SWNT H_2_O_2_ sensing studies that relied on simple corona designs (table S1). Using full-field seamless data captured through direct contact between the sensors and cells, this enables the monitoring of skin cell photoaging responses at various scales, from single-cell levels to clustered multiple-cell levels and makes continuous wave modeling and characterization possible because of the inherent continuity, in a label-free and real-time manner.

## RESULTS

### Synthesis and characterization of SNI

**Fig. 2.**
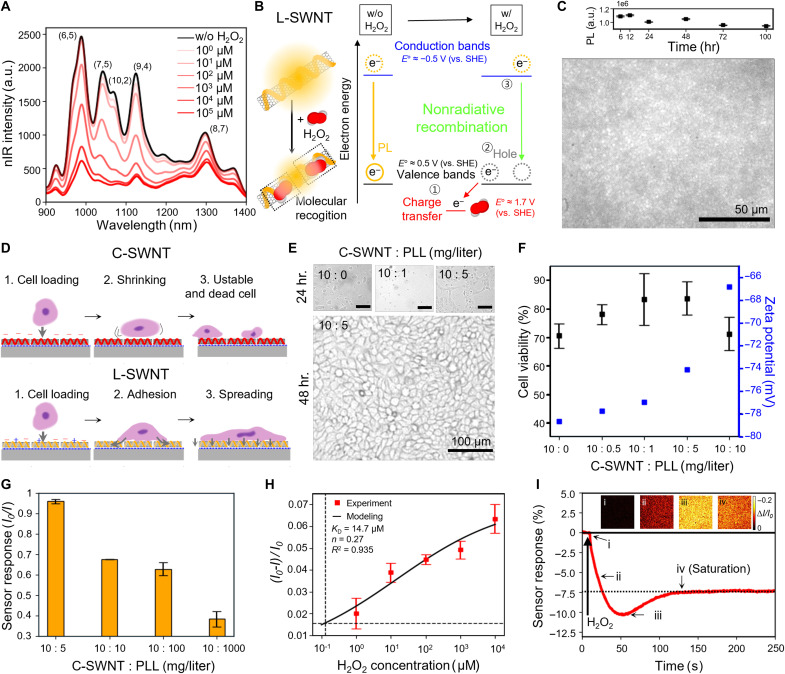
Synthesis and characterizations of SNI for label-free photoaging monitoring. (**A**) nIR fluorescence spectrum of L-SWNT to varying concentrations (1 to 10^5^ μM) of H_2_O_2_. (**B**) Schematic illustration of H_2_O_2_ molecular recognition mechanism of L-SWNT. (**C**) Time-series nIR intensity of L-SWNT (top) and representative nIR image of the nanosensor array (bottom). Scale bar, 50 μm. (**D**) Schematic illustration of skin cell adhesion metrics based on surface charge variations of C-SWNT and L-SWNT. (**E**) Bright-field images of cell growth at 24 hours (top) and 48 hours (bottom) after cell seeding across different PLL ratios. (**F**) Cell viabilities (black) and the corresponding zeta potential (blue) of the nanosensor array with varying PLL ratio. The data represent the mean value of *n* = 3 replicates. (**G**) Initial nIR fluorescence changes at different PLL ratios. The data represent the mean value of *n* = 3 replicates. (**H**) In vitro sensing response of the array with various concentrations of H_2_O_2_. The data represent the mean value of *n* = 3 replicates, and the intersection of the black dashed lines represents the LOD. (**I**) Real-time sensor response to 100 mM H_2_O_2_ and nIR images of specific time points (i, ii, iii, and iv) corresponding to the labeled points on the sensor response curve. The data represent the mean value of *n* = 3 replicates. hr., hours.

The synthesis of C-SWNTs was confirmed by UV-vis–nIR absorption spectra, which exhibited distinct peaks corresponding to the E_11_ and E_22_ transitions, indicating well isolation and suspension of individual SWNTs (fig. S1). nIR fluorescence measurements demonstrate that the corona phase of C-SWNTs enables high-sensitivity detection of H_2_O_2_, the final product of the type I photosensitization mechanism, with negligible response to ·O_2_^−^ compared to DNA-SWNTs synthesized with other pair-repeated DNA sequences (fig. S2). Furthermore, it also confirms its ability to detect H_2_O_2_ without interference from other biologically relevant oxidative molecules (fig. S3). In addition, the recovery of nIR fluorescence after the addition of catalase, a H_2_O_2_-decomposing enzyme, demonstrates that the signal we measure originates from H_2_O_2_ (fig. S4). Following PLL functionalization of C-SWNT, we measured the nIR responses of L-SWNT across a wide concentration range of H_2_O_2_ from 10^0^ to 10^5^ μM, which is a typical level released by cells under stress without triggering apoptosis. A dynamic and consistent decrease in the nIR signal was observed throughout the measurement (Fig. 2A). Each peak was assigned to specific chiralities based on previous researches (*35*, *36*), and all five major peaks exhibited concentration-dependent sensitivity to H_2_O_2_ following overall intensity variation (fig. S5). Figure 2B illustrates the H_2_O_2_-sensing mechanism of L-SWNT, where electron transfer from the E_11_ valence band of SWNT to the adsorbed H_2_O_2_ molecule driven by the redox potential difference between them, results in nonradiative recombination within the SWNT (*37*–*40*). This mechanism is further supported by the experimentally observed decrease in the E_11_ absorption peak (fig. S6). The calibration curve of L-SWNT estimated the limit of detection (LOD) to be 148 nM based on nIR intensity changes (fig. S7). Here, the LOD was calculated by adding the nanosensor response from the addition of only water as the noise level (σ) to three times the signal.

Photographs of the SNI show that L-SWNT is uniformly coated over whole surface of the glass substrate (fig. S8). nIR image of the SNI showed uniform and brightly emitting pixels on the glass substrate in high resolution (bottom, Fig. 2C). The pixel distribution data for all SNI samples used in this study exhibited high uniformity, with a marginal error of approximately 5% across the total brightness window (fig. S9). The L-SWNT maintained its average nIR fluorescence intensity with minimal variation (±10%) even over 100 hour, ensuring reliable signal mapping of skin cells even under long UV stress conditions (top, Fig. 2C). This nonphotobleaching characteristics of SWNTs is challenging to achieve with conventional dye-based ROS assays and imaging techniques (*23*, *41*–*43*). To optimize the ratio between C-SWNT and PLL for L-SWNT synthesis, we cultured keratinocytes on SNI with various PLL ratios. An nIR array with only C-SWNTs without PLL exhibits a highly negative surface charge, leading to unstable cell attachment due to the negatively charged cell membrane (top, Fig. 2D). In contrast, the L-SWNT array charge modified by PLL allows for stable cell spreading and consistent attachment (bottom, Fig. 2D). We cultured cells under the conditions of 10:0, 10:1, and 10:5 mg/liter (C-SWNT:PLL). Bright-field images after 24 hours of loading showed that cells cultured under the 10:5 condition exhibited optimum spreading on SNI. Furthermore, the cells displayed notable growth after 48 hours, effectively covering the entire frame (Fig. 2E and fig. S10). Zeta potential measurements showed that as the concentration of PLL increased, the surface negative charge of C-SWNT became less negative as we expected (Fig. 2F). Cell viability was evaluated using a commercially available colorimetric cell viability assay kit, demonstrating that the L-SWNT SNI at a 10:5 ratio exhibited the highest cell viability. This result aligns with the point where PLL only was shown to enhance cell viability (fig. S11). The decrease in cell viability at the 10:10 ratio might result from increased surface roughness by L-SWNT aggregations due to the high PLL concentration, as indicated by dynamic light scattering (fig. S12). This ratio also showed the least change in the baseline of the nIR fluorescence of L-SWNTs, both in the nIR spectra (Fig. 2G) and in the SNI form (fig. S13).

In vitro H_2_O_2_ response [(*I*_0_ − *I*)/*I*_0_] of SNI were measured by dropping H_2_O_2_ solutions onto the substrates, with the LOD determined to be 129 nM (Fig. 2H). Here, *I*_0_ and *I* represent the average nIR intensity of SNI area during the 10 s before H_2_O_2_ addition and 10 min after addition, respectively. SNI exhibited a consistent decrease in nIR signal by 1 to 7%, depending on the H_2_O_2_ concentration. Time series raw data and the corresponding brightness changes of individual pixels at each sample are shown in fig. S14. The interaction between the nanosensor and H_2_O_2_ can be modeled as first-order reversible reaction with the equilibrium constant (*K_A_*). Turn-off response of the nanosensor is proportional to the ratio of occupied binding sites (*A*θ) to total binding sites (θ_total_) can be described as follows (*44*)

$$\frac{I_{0}-I}{I_{0}}=\alpha \frac{\left[A\theta \right]}{\left[\theta_{\text{total}}\right]}=\alpha \frac{{\left(\left[A\right]K_{A}\right)}^{n}}{{\left(\left[A\right]K_{A}\right)}^{n}+1}\;\left(\text{with}\;K_{A}=\frac{\left[A\theta \right]}{\left[A\right]\left[\theta \right]}\right)$$ (1)

Fitting the data from Fig. 2H to Eq. 1 yielded a coefficient of determination (*R*^2^) value of 0.935, with a proportionality factor α of 0.07. The equilibrium dissociation constant (*K*_d_) was determined to be 14.7 μM, corresponding to 1/*K_A_*. The cooperativity of the binding reaction, indicated by *n* = 0.27, suggests negative cooperativity (*n* < 1), consistent with previous reports (*45*, *46*). Representative real-time nIR response data show that the SNI exhibited a 10% signal decrease within 50 s, followed by a slight recovery after the peak, reaching a steady state after 120 s (Fig. 2I). This immediate response of SNI enables the prompt reporting of H_2_O_2_ efflux from the overlying skin cells. We evaluated the reversibility of the SNI by applying 100 mM H_2_O_2_ three times, followed by a retrieving process, and observed a rapid return to baseline (fig. S15). These results confirm the suitability of our SNI for repetitive applications.

### Real-time monitoring and kinetic analysis of photoaging using SNI

**Fig. 3.**
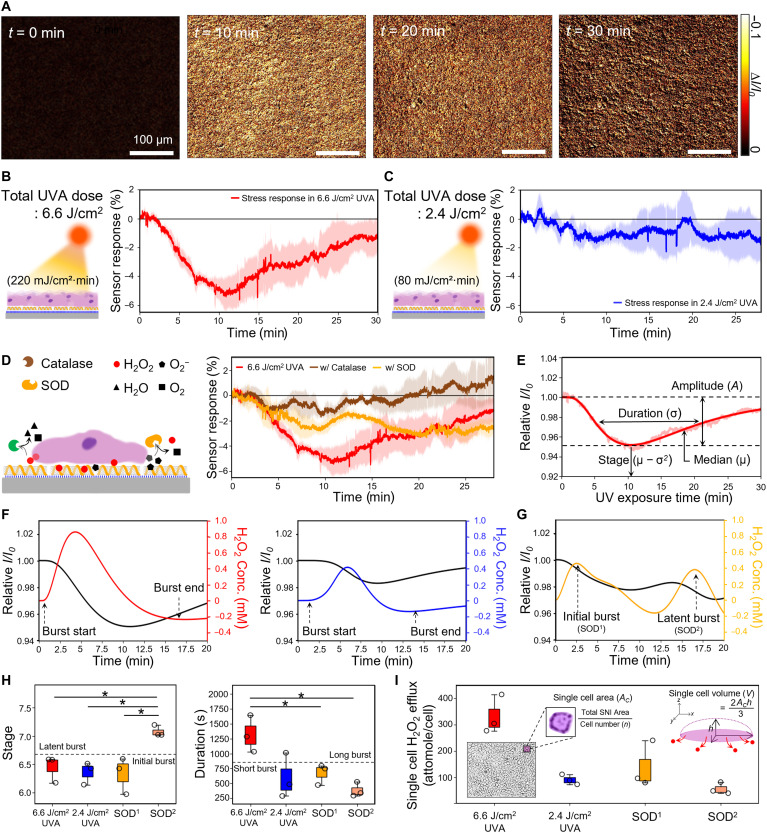
Real-time monitoring and kinetic analysis of photoaging using SNI. (**A**) Real-time nIR images of skin cells on the nanosensor array with UVA irradiation at 10 min intervals. Real-time sensor responses of the nanosensor array (**B**) under 6.6 J/cm^2^ UVA (220 mJ/cm^2^·min, 2.1 DER) and (**C**) 2.4 J/cm^2^ UVA (80 mJ/cm^2^·min, 0.77 DER). (**D**) Schematic illustration of the ROS scavenging mechanisms of catalase and SOD (left) and real-time sensor responses with the enzymes (right). For (B) to (D), the solid line represents the mean and the shaded region indicates σ from *n* = 3 biological replicates. (**E**) Representative fitting result (red line) and metrics of the log-normal probability density function model applied to the average time profile data. Real-time sensor responses with fitting (black) and H_2_O_2_ concentrations derived from the model (red) under (**F**) 6.6 J/cm^2^ UVA (left), 2.4 J/cm^2^ UVA (right), and (**G**) SOD treatment. (**H**) Differentiation of the burst stage (left) and duration (right) based on metrics from the fitted function. Two-tailed unpaired *t* tests: *P < 0.05. (**I**) Calculated H_2_O_2_ efflux from a single cell at the time of maximum burst. For (F) to (I), the data represent the mean from individual modeling of *n* = 3 independent biological replicates.

To monitor photoaging, we exposed daily UVA to keratinocytes grown on the SNI using a 365-nm UV lamp. A photograph of the detailed setup, along with the UVA lamp’s spectral and irradiance information, is shown in fig. S16. The total UV radiation on earth generally reaches 150 J/cm^2^ per day, with a UVA-to-UVB ratio of 27:1 (*47*), resulting in a daily exposure rate (DER) of 104 mJ/cm^2^·min. Of this, around 10% reaches human skin during daily activities (*22*, *48*), with UVA exposure estimated at 14.5 J/cm^2^. Therefore, we set the artificial UVA exposure intensity to match 2.4 J/cm^2^ UVA (80 mJ/cm^2^·min, 0.77 DER) and 6.6 J/cm^2^ UVA (220 mJ/cm^2^·min, 2.1 DER), accounting for typical altitude (*49*) and geographical factors (*47*) to simulate daily photoaging. Real-time nIR snapshots clearly showed an instantaneous quenching of the reporter pixels across the entire SNI area under 6.6 J/cm^2^ UVA, demonstrating real-time photoaging signals triggered by H_2_O_2_ stress waves from keratinocytes (Fig. 3A). Real-time nanosensor responses calculated from the movie show that the SNI detected a maximum H_2_O_2_ burst at around 10 min (−5.6%), followed by a gradual recovery over the 30-min UV exposure period (Fig. 3B). Under 2.4 J/cm^2^ UVA, the keratinocytes exhibited a weaker response of −1.7%, approximately 3.3 times less intense than under 6.6 J/cm^2^ UVA condition, and showed a sustained H_2_O_2_ release (Fig. 3C). In addition, we validated the H_2_O_2_ release pattern using the photometric hydrogen peroxide assay kit, which showed a peak concentration about fourfold lower and 5 min delayed compared to the nanosensor (fig. S17), likely due to the assay detecting bulk H_2_O_2_ after diffusion and degradation, whereas the nanosensor captures it directly at the cell-sensor interface. Furthermore, we confirmed that the observed H_2_O_2_ release is not caused by cell death from UV exposure but is instead associated with the photoaging process (fig. S18). These confirm that even daily UVA exposure induces the oxidative stress, which was detected owing to the sensitivity of the SNI. The full recorded nIR movies for each condition are shown in movies S1 and S2, respectively. The human epidermis is composed of keratinocytes and melanocytes in a 9:1 ratio (*50*). To demonstrate whether the photoaging kinetics we observed could be applied to a more realistic skin model, we monitored the SNI with a 10% melanocyte coculture, showing nearly identical sensing behavior with keratinocyte only skin model (fig. S19).

To confirm the immediate changes in the H_2_O_2_ wave in response to external environmental changes, we pretreated the skin cells with two key antioxidant enzymes involved in the ROS pathway including catalase and superoxide dismutase (SOD). Catalase is an enzyme in the ROS pathway that catalyzes the decomposition of H_2_O_2_ into water and oxygen, thereby reducing oxidative stress (left, Fig. 3D). SOD catalyzes the dismutation of O_2_^−^ into H_2_O_2_ and oxygen, playing a key role in mitigating oxidative damage. Nanosensor responses were largely suppressed by catalase, effectively serving as a negative control, confirming that the SNI accurately detects H_2_O_2_ efflux from keratinocytes (right, Fig. 3D). In contrast, SOD treatment led to a distinctive H_2_O_2_ efflux wave, characterized by an initial weakened burst followed by a rapid recovery and a secondary burst. This distinct enzyme-driven pathway control further confirms that the SNI specifically senses real-time H_2_O_2_ efflux.

To quantify the characteristics of the H_2_O_2_ wave, we modeled the real-time SNI signals using a log-normal probability density function to represent the temporal dynamics of the H_2_O_2_ wave (Eq. 2)

$$I\left(t\right)/I_{0}=1-\frac{A}{t\sigma \sqrt{2\pi }}\text{exp}\left(-\frac{{\left(\text{ln}t-\mu \right)}^{2}}{2\sigma^{2}}\right)$$ (2)

By fitting the data from Fig. 3B to Eq. 2, we obtained a high correlation with *R*^2^ = 0.976, yielding three key parameters: *A* = 59.1, μ = 6.85, and σ = 0.64 (Fig. 3E). The proportionality factor *A* can represent the amplitude of the wave, indicating the overall intensity of H_2_O_2_ burst. The mode of the distribution (*e*^μ*−*σ*2*^) can be translated to the wave stage at which the burst occurs. Last, the duration of the burst can be determined by evaluating the value of e^μ^ (e^σ^ − e^−σ^). To further quantify the nIR intensity as the actual spatial H_2_O_2_ concentration, we developed an adsorption model based on a first-order reversible reaction following differential equation

$$\begin{array}{c} \frac{d\left[H_{2}O_{2}-\text{SWNT}\right]}{dt}=k_{f}\left[H_{2}O_{2}\right]\left[\text{SWNT}\right]-k_{r}\left[H_{2}O_{2}-\text{SWNT}\right] \end{array}$$ (3)

where *k*_f_ means the forward reaction constant and *k*_r_ means the reverse reaction constant (*51*). Assuming that the total amount of SWNTs in the array space remains constant and considering the quenching reaction of H_2_O_2_ with SWNTs, the local concentration of H_2_O_2_ at the specific time (*t*) on the array is

$${\left[H_{2}O_{2}\right]}_{t}=\frac{k_{r}\left[1-I\left(t\right)/I_{0}\right]-d\left[I\left(t\right)/I_{0}\right]/dt}{k_{f}\times I\left(t\right)/I_{0}}$$ (4)

Here, the ratio between *k_x_* and *k_r_* was derived from the previously determined *K*_d_, and *k*_f_ and *k*_r_ were subsequently calculated to be 0.178 M^−1^ s^−1^ and 2.6 μs^−1^, respectively, based on in vitro measurements performed on the SNI platform (fig. S20). These experimentally derived values are expected to reflect surface adsorption and diffusion characteristics under the sensing conditions of SNI. Detailed model derivations are provided in the note S1.

Translated H_2_O_2_ concentration profiles based on the models revealed that the keratinocytes began the stress burst at 54 s and released a maximum of 857 μM at 257 s under approximately twice the intensity of the DER, with the burst concluding at 1042 s (left, Fig. 3F). In contrast, under 2.4 J/cm^2^ UVA conditions, the burst began later at 99.5 s, with the maximum release of 437 μM at 354 s, and the burst concluding earlier at 794 s (right, Fig. 3F). In the case of SOD pretreatment, the enzymatic activity led to a split into two distinct bursts under 6.6 J/cm^2^ UVA. The initial burst (SOD^1^) began at 10 s, showing an H_2_O_2_ efflux of 450 μM, followed by a latent burst (SOD^2^) starting at 783.5 s with a reduced efflux of 355 μM (Fig. 3G). We set a stage index (μ *−* σ*^2^*) of 6.62 (corresponding to e^6.62^ = 750 s) as the threshold, defining values below this as indicative of an initial burst and values above as a latent burst. In addition, we set a duration index [e^μ^ (e^σ^ − e^−σ^)] threshold at 800 s, categorizing long and short bursts. On the basis of these evaluation metrics, the 6.6 J/cm^2^ UVA, 2.4 J/cm^2^ UVA, and SOD^1^ conditions were classified as initial responses with stage indices of 6.45, 6.36, and 6.33, respectively, while SOD^2^ was identified as a delayed response with a stage index of 7.08. The duration indices for these conditions were 1312, 594, 670, and 384 s, respectively, demonstrating that the burst under 6.6 J/cm^2^ UVA uniquely qualifies as a long burst (Fig. 3H). The SOD waveform might be attributed to the conversion of ·O_2_^−^, the primary product in the ROS pathway (*52*, *53*), into H_2_O_2_ due to the externally added SOD. This early conversion might reduce oxidative stress, resulting in a decreased intensity of the initial burst (*54*, *55*). The residual H_2_O_2_, while less reactive than ·O_2_^−^, could have subsequently induced a weaker oxidative stress (*56*, *57*), leading to a shorter and less intense latent burst.

We calculated the H_2_O_2_ efflux at single-cell level by defining the volume of a single keratinocyte and cell numbers on SNI (detail calculations are shown in note S2) (*58*). Results indicate that the maximum H_2_O_2_ efflux was quantified as 432, 190, 238, and 156 attomoles per single keratinocyte for the respective conditions (Fig. 3I). In addition, we obtained the wave curves normalized to the kinetics of H_2_O_2_ release from a single cell (fig. S21). On this basis, under the respective conditions, a single skin cell released H_2_O_2_ at an early burst rate of 132, 58.5, 90.6, and 43.9 attomole/cell·min. These quantitative characterizations of the photoaging wave are made possible by real-time and label-free sensing capabilities of our SNI.

### Spatiotemporal analysis of H_2_O_2_ waves during photoaging

**Fig. 4.**
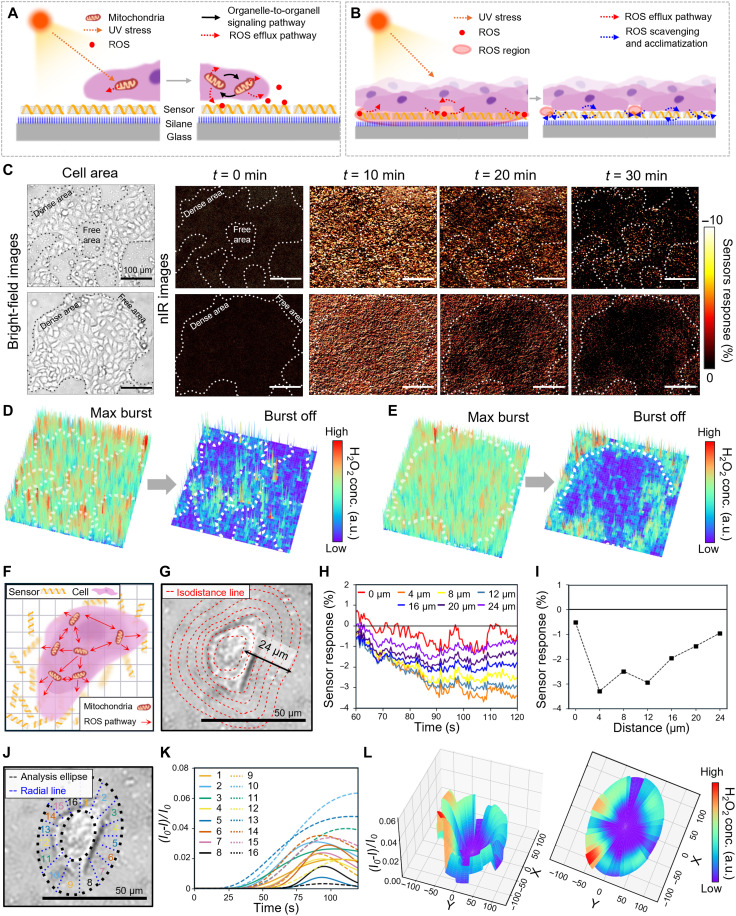
High spatiotemporal precision analysis of H_2_O_2_ waves during photoaging. Schematic illustration of (**A**) the intracellular stress response and (**B**) stress signaling and acclimatization. (**C**) Bright-field image of sparsely grown cells (left) and nIR images at 10 min-intervals (right) for analyzing spatial H_2_O_2_ distribution during the photoaging under 6.6 J/cm^2^ UVA. 3D spatial nIR response map at the time of max burst (*t* = 10 min) and burst off (*t* = 30 min) from the data from (**D**) top of (C), and (**E**) bottom of (C). (**F**) Schematic illustration of the intracellular stress response and ROS pathway within a single cell on the nanosensor array. (**G**) Bright-field image of a single keratinocyte with designated regions, defined by isodistance lines (red) set at 4-μm intervals from the cell core. (**H**) Real-time nIR sensor responses and (**I**) the mean response magnitude at 110 to 120 s from the designated regions at varying distances. (**J**) Bright-field image with a designated elliptical region division into 16 equal sections by radial lines (blue). (**K**) Fitted time-series sensor change rate represented by the colors of the defined 16 sections. (**L**) 3D graphs of H_2_O_2_ efflux from a single keratinocyte with elevated view (left) and top view (right).

Next, we analyzed the stress signaling and acclimatization behaviors at both single-cell and multicellular resolutions using a high-resolution nIR imaging and 3D signal mapping (*59*–*61*). When cells experience photo-stress, immediate organelle-to-organelle ROS signaling occurs at the intracellular level before cell-to-cell communication (*62*–*64*). Specifically, the increase in intracellular Ca^2+^ level leads to the collapse of the mitochondrial membrane potential (Δψ*_m_*), which triggers the generation of ROS within the mitochondria (*65*), known as mitochondrial flashes (*66*–*68*) (mitoflashes) (Fig. 4A). To confirm the intracellular mitoflashes, we double-loaded the skin cells with tetramethylrhodamine ethyl ester perchlorate (TMRE), a Δψ*_m_*-sensitive dye, and DCF-DA, an intracellular ROS indicator (*69*). Confocal microscope images clearly demonstrated an immediate collapse of Δψ*_m_* followed by a gradual recovery, alongside intracellular ROS formation peaking between 6 and 9 min (fig. S22). It proves that intracellular events occur slightly earlier than the extracellular responses detected by the SNI. When mitoflash occurs within a single cell, the cell responds by releasing H_2_O_2_ through aquaporin 3 as part of its cell-to-cell signaling (*70*). Initially, this leads to the formation of an ROS-rich region across the cell growth area (*71*). However, under sublethal stress conditions, cells activate oxidative stress regulation mechanisms and lead to acclimatization (*72*), specifically called photoadaptation (Fig. 4B) (*73*).

To monitor this photoadaptation behavior, we exposed 6.6 J/cm^2^ UVA to distinctly populated cell area on SNI (Fig. 4C). No movement of the cell populations was observed during the experiments, confirming that all quenching signals originated from photoadaptation (fig. S23). A maximum H_2_O_2_ burst was observed within 10 min, consistent with previous results in Fig. 3A (fig. S24). However, after this peak, the H_2_O_2_ response began to diminish from the center of the cell-dense area, and after 30 min, the H_2_O_2_ detection response had mostly vanished in these regions. Notably, when cells grew densely around a central area, a more pronounced acclimatization response was observed compared to cells that were more sparsely distributed or interconnected with gaps in between (movies S3 and S4). This phenomenon might be attributed to the necessity for closely positioned gap junctions between cells (*74*), which serve as crucial channels for effective paracrine signaling during the process of maintaining organismal homeostasis (*75*). 3D signal maps based on nIR imaging revealed these features more distinctly (Fig. 4, D and E). Figure 4F illustrates the stress response mechanism to photoaging at the single-cell level in keratinocytes, derived from our measured data.

To further investigate site-specific H_2_O_2_ efflux induced by photoaging, we performed high-resolution (100× magnification) analyses of efflux patterns in single keratinocyte cells. First, the cell boundary was divided into 4-μm segments, and the average response within each area was calculated (Fig. 4G). H_2_O_2_ responses were detected starting at approximately 60 s displaying distinct patterns based on the distance from the cell core (Fig. 4H). The calculated nIR sensor responses were highest at the membrane boundary (4 μm from the core) and gradually decreased in intensity with increasing distance, extending up to 24 μm, which corresponds to the cell’s diameter (Fig. 4I). Then, we assumed the cell to have an elliptical shape and subdivided the analysis region into 16 sections using radial lines spaced at uniform angles (∅) to examine the anisotropic characteristics of H_2_O_2_ wave (Fig. 4J). Sensor responses in each section (*i*) were fitted to Eq. 2, revealing stress levels that varied considerably, with differences of up to 20.2-fold between directions (Fig. 4K). To spatially visualize the time-dependent H_2_O_2_ efflux of the cell, we transformed the *x*-*y* plane of the cell into a polar coordinate system defined by ∅ and time (*t*), mapping the sensor responses to the *z* axis using the model

$$z\left(\phi ,t\right)=\frac{A_{i}}{t\sigma_{i}\sqrt{2\pi }}\text{exp}\left(-\frac{{\left(\text{ln}t-\mu_{i}\right)}^{2}}{2\sigma_{i}^{2}}\right)$$ (5)

where

$$\phi \in \left[\frac{\left(i-1\right)\cdot 2\pi }{16},\frac{i\cdot 2\pi }{16}\right),i=1,2,\ldots ,16$$ (6)

The results clearly demonstrate a notable anisotropic H_2_O_2_ wave variation depending on the polar angle (Fig. 4L). The raw sensor responses for each section are provided in fig. S25. Notably, when matching the efflux profile with bright-field image, efflux was minimal at the focal adhesion sites of the cell (e.g., sections 1–2 and 8–9) demonstrating a link between the growth direction of skin cells and photoaging. The same trend was observed in the other single cell (fig. S26). More details on the 3D modeling process including parameter definition, along with the corresponding data, are provided in note S3. In addition, in a lower magnification (10×), where keratinocytes densely populate a broader area, the same stress response was observed (fig. S27). Through spatiotemporal monitoring of the photoaging process using SNI under various scaling conditions and keratinocyte distributions, we observed that single skin cells exhibit anisotropic characteristics in the release of H_2_O_2_, with directional propagation from the center to the periphery, specifically originating from regions not associated with their growth zones. Multicellular structures, in turn, use the extracellularly released H_2_O_2_ as a signaling molecule to coordinate stress communication and photoadaptation responses across the cell cluster.

### SNI as a label-free platform for cosmetic antioxidant evaluation

**Fig. 5.**
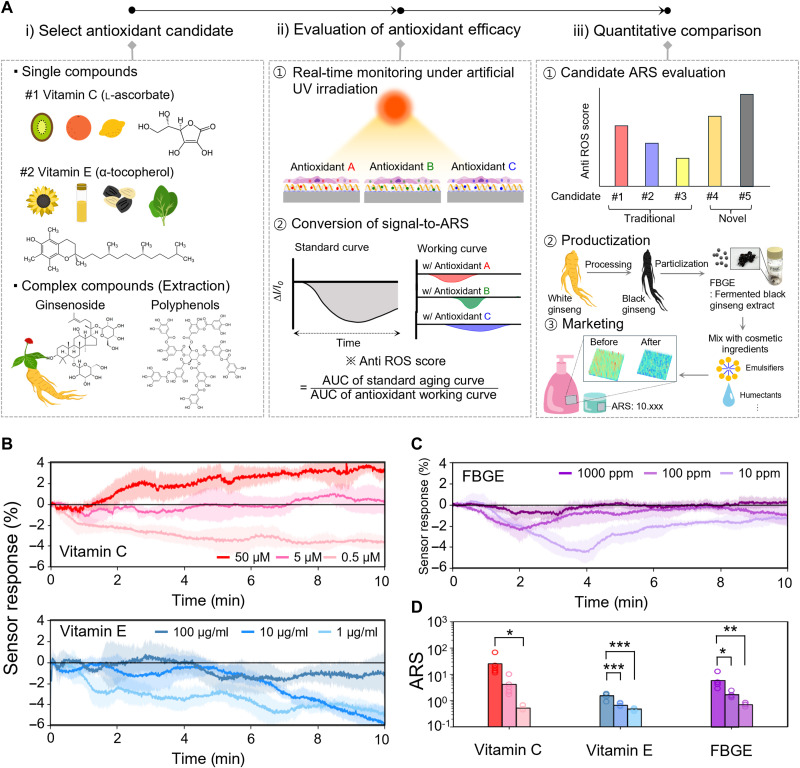
SNI as a label-free platform for evaluating antioxidant performance of the materials. (**A**) Conceptual workflow for evaluating the antioxidant efficacy of cosmetic ingredients against photoaging leveraging the nanosensor array. Real-time photoaging responses of the nanosensor array with pretreatment of the cosmetic ingredients including (**B**) 0.5, 5, and 50 μM vitamin C (top); 1, 10, 100 μg/ml vitamin E (bottom); and (**C**) 10, 100, 1000 ppm FBGE. The mean value is shown as a solid line, with the shaded area representing σ across *n* = 5 biological replicates. (**D**) ARS calculated from the real-time measurements for each ingredient. One-way analysis of variance (ANOVA) followed by Tukey’s HSD post hoc test. *P < 0.05, **P < 0.01, ***P < 0.001.

By leveraging the established skin cell photoaging model, we demonstrated that SNI can be used as a label-free platform for the quantitative evaluation of antioxidant performance as an example application (Fig. 5A). This platform addresses the need for methods to measure antioxidant performance under daily UV exposure while also allowing for quantitative comparison of product efficacy and spatial antioxidant distribution in the skin (*76*, *77*). The first step is to select candidate antioxidants for cosmetic products. As an example, we selected representative singular compounds such as vitamins C and E, as well as plant extracts such as ginseng, which exhibit potent antioxidant effects due to the synergistic interaction of active components such as ginsenosides and polyphenols (*78*). Then we evaluate the antioxidant effects of the materials using SNI. To assess their efficacy after stabilization within the skin, a 24-hour pretreatment is applied before monitoring, ensuring that the substances do not interfere with the sensor signal. nIR curves of each candidate are collected, and their area under the curve (AUC) ratio based on the standard curve are calculated. We termed this evaluation metric as anti-ROS score (ARS)

$$\mathrm{ARS}=\frac{\mathrm{AUC}\;\text{of standard aging curve}}{\mathrm{AUC}\;\text{of antioxidant working curve}}$$ (7)

A higher ARS indicates greater antioxidant efficacy, while an ARS close to or below 1 suggests reduced effectiveness or potential adverse effects under photo-stress. Therefore, ARS values enable a quantitative comparison of the antiaging performance of each candidate. This process allows for the accelerated product development of high-ARS materials selected for their superior performance in the cosmetic industry, while the SNI-recorded movie can be effectively used for visual marketing.

We used this platform to analyze a commercial antioxidant cosmetic product containing fermented black ginseng extract (FBGE). Detailed product information is shown in fig. S28. Various doses of vitamin C (0.5, 5, and 50 μM) and vitamin E (1, 10, and 100 μg/ml) were included as comparison groups to benchmark against representative cosmetic ingredients. These concentrations were selected considering their physiological plasma levels (*79*, *80*) and their effectiveness in mitigating UV-induced oxidative stress, specifically in keratinocytes (*81*, *82*). In addition, it was confirmed that antioxidants at this concentration have negligible impact, remaining within the noise window of the nIR fluorescence of the SNI (fig. S29). In the standard curve, the sensor response gradually decreased to −3% over time (fig. S30), whereas all six vitamin pretreatment cases showed substantially reduced sensor responses (Fig. 5B). For 50 μM vitamin C, a short response of −0.9% was observed within 1 min, followed by a slight increase in response behavior similar to that observed during the detection of dehydroascorbic acid (DHA), a specific byproduct of vitamin C’s intracellular antioxidant activity through its reaction with H_2_O_2_ (fig. S31) (*83*, *84*). In contrast, 5 μM vitamin C exhibited a peak response of −1% within 6 min, with no further responses from either H_2_O_2_ or DHA detected beyond that time, whereas 0.5 μM vitamin C showed a general decreasing response reaching −4% over the initial 10 min. The vitamin E group showed distinct patterns: For high-dose (100 μg/ml) vitamin E, an initial burst (−0.9%) was followed by a secondary burst (−2.8%) and subsequent recovery. In contrast, medium-dose (10 μg/ml) vitamin E exhibited two minor bursts (−1 and −1.2%) during the early stage (~5 min) but failed to recover after the later, larger burst. Low-dose (1 μg/ml) vitamin E showed a burst response similar to the condition without vitamin E, with a continuous decrease reaching −6% without a distinct multistage pattern. The FBGE group displayed a prolonged initial burst over the 0 to 6–min range. Specifically, high-dose [1000 parts per million (ppm)] FBGE showed a peak response of −1.3% during the 1 to 3–min range, while medium-dose (100 ppm) FBGE reached a peak response of −2.0% during the 0 to 6–min range. Low-dose (10 ppm) FBGE exhibited a delayed but distinct peak response of −4.3% occurring later in the observation period, followed by partial recovery (Fig. 5C). These results indicate distinct temporal antioxidative activity patterns for each skin care ingredient. The full recorded nIR movie for FBGE’s antioxidative working condition is shown in movie S5. ARS values calculated from the SNI data were 25.96, 4.23, 0.55, 1.51, 0.70, 0.46, 5.89, 1.67, and 0.73 for the respective concentrations of vitamin C, vitamin E, and FBGE (Fig. 5D). Real-time raw data for five replicates of all samples are shown in fig. S32. FBGE (1000 ppm) demonstrated 3.9- and 1.4-times higher antioxidant performances than that of vitamin E (100 μg/ml) and vitamin C (5 μM). This workflow also enables 3D signal mapping for enhanced visual understanding of the antioxidant materials (fig. S33). Overall, this SNI provides a label-free platform to quantitatively assess and precisely compare the performance of various antioxidant materials without restriction while simultaneously enabling real-time monitoring of H_2_O_2_ efflux waves with high spatiotemporal resolution at the molecular level. These capabilities may inform the optimization of treatment parameters such as dosage, application timing, and target area, supporting the commercialization of products in therapeutic or cosmetic fields.

## DISCUSSION

In conclusion, we developed a label-free, real-time photoaging monitoring platform with high spatiotemporal resolution using a highly sensitive nIR fluorescent SWNT array. Through the dual functionalization of SWNTs with ssDNA and PLL, we achieved both H_2_O_2_ sensitivity and skin cell compatibility enabling simultaneous skin cell culture and nIR monitoring. Using SNI, we demonstrated a specific H_2_O_2_ burst peaking at around 10 min in response to even 2.4 J/cm^2^ UVA exposure (80 mJ/cm^2^·min) and quantified H_2_O_2_ concentrations as 274 μM under natural conditions. In addition, through numerical modeling, we characterized the amplitude, stages, and duration of distinct H_2_O_2_ waveforms across various photoaging conditions by using specific modeling parameters. Real-time nIR movie revealed distinct photoadaptation behavior in skin cells following UVA exposure, and single-cell H_2_O_2_ waves demonstrated a link between the direction of photoaging stress waves and regions outside the growth orientation of the cells, with an efflux rate of 132 attomole/cell·min. We demonstrated the application potential of SNI as a label-free, quantitative platform for evaluating antioxidant materials, specifically targeting the cosmetic field with direct relevance to daily photoaging, unlike previous SWNT-based H_2_O_2_ sensing studies that lacked real-world applications (table S1). We introduced the ARS metric using real-time ROS burst data to assess antioxidant performance and evaluate commercial cosmetic ingredients. This label-free and SNI allows for the unveiling of the initial onset of photoaging through real-time spatiotemporal monitoring of H_2_O_2_ release during continuous and unavoidable daily solar UV exposure. It can uncover the intricate biochemical pathways of photoaging, providing a foundation for the development of future biopharmaceutical and cosmetic products. Furthermore, given the central role of dysregulated ROS in biological processes such as inflammation, wound healing, and oxidative skin disorders, this sensing strategy holds further potential for broader biomedical applications beyond photoaging. While a 2D keratinocyte model may not directly capture the full complexity of photoaging across the 3D architecture of human skin, recent studies have demonstrated the physiological relevance and translational applicability of 2D epidermal models in comparison to 3D skin systems (*85*–*88*). In this context, the spatiotemporal information revealed here could serve as a key to understanding real-life photoaging dynamics in human skin.

## MATERIALS AND METHODS

### Synthesis and characterization of L-SWNT

Raw SWNTs (NanoIntegris), produced using the HiPCO process, were mixed with 28-base (CCCT)_7_ and pair-repeated 30-base ssDNA sequences—(GT)_15_, (AT)_15_, (CT)_15_, and (CC)_15_ (Integrated DNA Technologies)—in a 1:1 mass ratio using deoxyribonuclease (DNase)–free water. The mixture was bath sonicated (WUC-D03H, Daihan Scientific) for 10 min and additionally ultrasonicated by a probe sonicator (Q125, QSonica) for 30 min using a 1/8″ (0.3175 cm) probe (Cole Parmer) in an ice bath. Subsequently, the sonicated dispersion was centrifuged (2 × 60 min, 1730R, Gyrozen) at 17,000 relative centrifugal force to remove aggregates. The supernatant yielded homogenously dispersed C-SWNT, and the concentration was adjusted to 10 mg/liter using UV-Vis–nIR absorption spectroscopy (UV-2600i, Shimadzu), using an extinction coefficient of ε_632nm_ = 0.036 mg/liter·cm. L-SWNT was prepared by mixing a DNase-free water-diluted PLL solution (P8920, Sigma-Aldrich) with C-SWNT (10 mg/liter) at a 1:1 volume ratio. The hydrodynamic size, stability, and surface charge of the nanosensors were measured using zetasizer (ZS90, Malvern Instruments). The sensor fluorescence was monitored under 761-nm laser excitation at 300 mW (MRL-FN-721, CNI Optics) before and after the addition of analytes using automated nIR spectroscopy (IX73, Olympus), with the system comprising an inverted microscope with a 20× nIR objective, paired with an nIR spectrometer (SpectraPro HRS 300S, Princeton Instruments) and an InGaAs 1D detector (PyLoN-IR, Princeton Instruments). Twenty microliters of various concentrations of H_2_O_2_ solution (H1009, Sigma-Aldrich) was added to 180 μl of C-SWNT sensor solution, making a total volume of 200 μl in a 96-well plate, under both DNase-free water and phosphate-buffered saline (PBS, pH 7.4, Life Technologies) buffered conditions. After incubating at room temperature for 30 min, the fluorescence intensity of three replicates was measured. For additional ROS screening, superoxide (·O_2_^−^) was generated by the reaction between 300 μM xanthine (X0626, Sigma-Aldrich) and xanthine oxidase (10 mU/ml; X4379, Sigma-Aldrich); singlet oxygen (^1^O_2_) was produced by reacting 100 μM CuCl_2_ (C3279, Sigma-Aldrich) with 100 μM H_2_O_2_; and hydroxyl radicals (·OH) were generated through the reaction between 100 μM FeSO_4_ (F7002, Sigma-Aldrich) and 100 μM H_2_O_2_. The change in fluorescence intensity was measured by comparing the C-SWNT response before and after the addition of xanthine and a metal cation, followed by a 30-min incubation, with the intensity measured after the subsequent addition of xanthine oxidase. For nitrogen species, 100 μM of NaNO_2_ (S2252, Sigma-Aldrich) and NaNO_3_ (S5506, Sigma-Aldrich) were added for NO_2_^−^ and NO_3_^−^, respectively, and 100 μM of NH_4_Cl (A4514, Sigma-Aldrich) was added for NH_4_^+^. After a 30-min incubation, the change in fluorescence intensity was measured. For NO, 50 μM of the NO donor DEA NONOate (D5431, Sigma-Aldrich) was dissolved in a PBS-buffered solution containing 10 mM NaOH and immediately added to the C-SWNT solution, and the fluorescence intensity change was measured instantly. All intensity change measurements were performed in triplicate.

### Fabrication of SNI based on L-SWNT

Glass-bottom petri dishes (200350, SPL Life Sciences) were exposed to UV for 15 min (AC-3, AhTech LTS), followed by coating the glass surface with 40 μl of 1.5 wt % APTES/H_2_O in ethanol solution, and incubating at room temperature for 2 hours Subsequently, the petri dishes were rinsed with ethanol and immediately dried with N_2_ gas. Next, 250 μl of nanosensor solution was applied, uniformly covering the whole glass surface, and dried at room temperature for 12 hours To remove unbound nanosensor, the surface was smoothly rinsed with H_2_O and dried using N_2_ gas. To develop a cell-compatible surface, L-SWNT was prepared by mixing C-SWNT with PLL solution at various mass ratios, and the resulting mixture was then coated onto the surface using the above protocol.

### Skin cell cultures on SNI

The immortalized human keratinocyte cell line (HaCaT) and human melanocyte cell line (SK-MEL-28) were obtained from the Korean Cell Line Bank. Keratinocytes, melanocytes, and their cocultures were all maintained in Dulbecco’s modified Eagle’s medium (DMEM, LM001-05, Welgene) supplemented with 10% fetal bovine serum (S001-01, Welgene) and 1% penicillin-streptomycin (15140, Gibco) at 37°C in a humidified atmosphere containing 5% CO_2_. Keratinocytes and melanocytes were each seeded at a density of 3 × 10^4^ cells per dish in glass-bottom petri dishes coated with a designated concentration ratio of C-SWNT:PLL. For coculture experiments, keratinocytes were seeded at a density of 2.7 × 10^4^ cells and melanocytes at a density of 3 × 10^3^ cells per dish. Keratinocytes were subsequently cultured on these surfaces, and the optimal L-SWNT composition for cell viability was identified using a Cellomax viability assay (CM-VA0500, PRECARE GENE). After 24 hours of incubation, the cells were washed with PBS, and a viability assay solution diluted in DMEM was added to each well. Following 2 hours of incubation, the absorbance was measured at 450 nm using a microplate reader (Spark, Tecan, Switzerland). To measure extracellular H_2_O_2_ concentrations during the photoaging process, the cells were washed with PBS, and 100 μl of medium was collected every 5 min. Absorbance at 446 nm was then measured using a photometric hydrogen peroxide test kit with a micromolar detection range (118789, Merck Millipore). To examine the intracellular stress response during photoaging, keratinocytes were double-loaded with the intracellular ROS indicator DCF-DA (ab113851, Abcam) and the mitochondrial membrane potential indicator TMRE (87917, Sigma-Aldrich) at concentrations of 20 and 100 nM, respectively, 15 min before UV irradiation. After 15 min, both fluorescent dyes were replaced with phenol red–free DMEM. During the photoaging process, DCF-DA and TMRE fluorescence were observed using confocal microscopy (TCS SP8 HyVolution, Leica Microsystems CMS GmbH) at excitation wavelengths of 529 and 595 nm, respectively, with 20× magnification, and images were captured at 3-min intervals.

### nIR imaging of SNI for photoaging monitoring

Spatial nIR imaging of the nanosensor interface was conducted using nIR microscopy. The light from a 721-nm laser was expanded via a beam expander and then reflected by a beam splitter. For each experiment, the light passed through a selected objective lens with a specific magnification, exciting the surface coated L-SWNT and inducing nIR emission. The emitted nIR fluorescence was filtered through a 900-nm long-pass filter and detected by an InGaAs camera (NINOX 640 II, Raptor Photonics; 14-bit output). For analysis, the signal was rescaled to an 8-bit range (0–255), where the measured dark noise was 7 × 10^−5^% and the long-term baseline shift was 6 × 10^−3^% (fig. S34). This setup enabled the collection of nIR fluorescence images and movies at various scales using different objective lenses across experiments. A 500 ms of exposure time was used for 10× and 20× magnifications, while a 1-s exposure time was required for 100× magnification due to lower light intensity. To validate the H_2_O_2_ detection capability of the coated phase nanosensor interface, 300 μl of DMEM was applied to cover the circular coated area uniformly immediately after fabrication, and the petri dishes were incubated for 24 hours to equilibrate the nanosensor under the cellular environment. Subsequently, 30 μl of various concentrations of H_2_O_2_ solution were gently applied, and the spatial variations in fluorescence intensity were recorded over a 10-min period in triplicate. To induce artificial photoaging while excluding other stimuli in the presence of DMEM, the cells were irradiated with 365-nm UVA using a UV Handy Lamp (VL-215 L, Vilber Lourmat). The distance between the UV lamp and the cells was adjusted according to the inverse-square law to control UVA power, which was measured using a power meter (PM100D, Thorlabs), with irradiation conducted at distances of 12 cm (220 mJ/cm^2^∙min) and 20 cm (80 mJ/cm^2^∙min) for about 30 min. Simultaneously, spatial nIR fluorescence changes corresponding to the cellular response were recorded in real-time with a 500-ms exposure time by nIR camera. To assess the presence of H_2_O_2_ and temporally monitor enzymatic activities under photoaging conditions, 200 μl of catalase (500 U/ml; C9322, Sigma-Aldrich) and SOD (300 U/ml; S5395, Sigma-Aldrich) were each pretreated for 30 min before UV irradiation. Subsequently, the cells were exposed to UVA at an intensity of 220 mJ/cm^2^·min for about 30 min.

### Image analysis for signal calculations

Image and data analysis were performed using Python’s OpenCV library and Fiji. To obtain a time profile of the sensor response upon photoaging, a UV signal subtraction process was first performed to eliminate interference from the UV lamp on the nIR camera. The average area pixel intensity was calculated for 10 s before and after the UV lamp was turned on, and the difference was used to subtract the UV signal detected by the nIR camera. Following this correction, the *I*_0_ was established as the average sensor intensity for the 10-s period spanning ±5 s from the time the UV lamp was activated. The sensor response was then calculated as the percentage change in intensity, defined by (*I − I*_0_)/*I*_0_, where *I* is the sensor intensity at any given time after the baseline period.

### Evaluation of cosmetic antioxidant reagents

Ascorbic acid and (+)-α-tocopherol acetate were purchased from Sigma-Aldrich. FBGE was provided by Shinsegae International as a component of YUNJAC ALPHANAX. To simulate the absorption of cosmetic antioxidant reagents in skin cells and to ensure that they do not interfere with H_2_O_2_ detection by the nanosensor interface, four separate experiments were conducted. In each experiment, six antioxidant concentrations were tested: 50 and 5 μM ascorbic acid, (+)-α-tocopherol acetate (100 and 10 μg/ml), and 1000 and 100 ppm FBGE. Each concentration was diluted in 2 ml of DMEM and pretreated 24 hours after the initial cell seeding. The cells were then incubated with the respective reagent for an additional 24 hours. For DHA screening, 50 μM (L)-DHA (261556, Sigma-Aldrich), dissolved in DMEM, was gently applied, and spatial variations in fluorescence intensity were recorded over a 10-min period, with measurements performed in triplicate.

## Supplementary Materials

### The PDF file includes:

Supplementary Notes S1 to S3 Figs. S1 to S34 Table S1 Legends for movies S1 to S5 References
