# Supplementary material for: Label-free and real-time monitoring of photoaging with high spatiotemporal resolution using an nIR fluorescent nanosensor array
Source: Sci Adv. 2025 Sep 12;11(37):eadt2296. doi: 10.1126/sciadv.adt2296 (PMC12429032; doi:10.1126/sciadv.adt2296)
Supplement: Supplementary file 1 — Supplementary Notes S1 to S3 Figs. S1 to S34 Table S1 Legends for movies S1 to S5 References [file sciadv.adt2296_sm.pdf]

Science Advances
AAAS

Supplementary Materials for

Label-free and real-time monitoring of photoaging with high spatiotemporal resolution using an nIR fluorescent nanosensor array

Youngwook Cho *et al.*

Corresponding author: Soo-Yeon Cho, sooyeonc@skku.edu

Sci. Adv. **11**, eadt2296 (2025)
DOI: 10.1126/sciadv.adt2296

The PDF file includes:

Supplementary Notes S1 to S3
Figs. S1 to S34
Table S1
Legends for movies S1 to S5
References

Other Supplementary Material for this manuscript includes the following:

Movies S1 to S5

Supplementary Notes.

Supplementary Note S1. Reaction kinetics modeling between SNI and H_2O_2.

The response of the nanosensor array is expressed as a time-dependent continuous function, where the amount of H_2O_2 present in the spatial domain of the array can be quantified according to a first-order reversible reaction[46]. The process of H_2O_2 adsorption and desorption on the nanosensor array, using the forward reaction constant (k_f) and reverse reaction constant (k_r), is modeled by:

$$\mathrm{SWNT} + \mathrm{H_2O_2} \underset{k_r}{\overset{k_f}{\rightleftarrows}} \mathrm{H_2O_2} - \mathrm{SWNT} \quad \text{(S1)}$$

The reaction rate can be expressed by the following differential equation:

$$\frac{\mathrm{d}[\mathrm{H_2O_2{-}SWNT}]}{\mathrm{d}t} = k_f[\mathrm{H_2O_2}][\mathrm{SWNT}] - k_r[H_2O_2 - SWNT] \quad \text{(S2)}$$

In the quenching reaction of H_2O_2 with SWNTs, the relative sensor signal (I/I_0) is proportional to the amount of unoccupied SWNTs. In terms of the ratio, this can be expressed as:

$$I/I_0 = [\mathrm{SWNT}]/[\mathrm{SWNT}]_0 \quad \text{(S3)}$$

, where $[\mathrm{SWNT}]_0$ represents the initial amount of SWNTs present in the array. Assuming that the total amount of SWNTs in the array remains constant, the proportion of H_2O_2-occupied SWNTs can be expressed as follows:

$$[\mathrm{SWNT}]_0 = [\mathrm{SWNT}] + [\mathrm{H_2O_2} - \mathrm{SWNT}] \quad \text{(S4)}$$

$$[\mathrm{H_2O_2} - \mathrm{SWNT}]/[\mathrm{SWNT}]_0 = (I_0 - I)\ /\ I_0 \quad \text{(S5)}$$

By substituting Supplementary Eq. (S3) and Eq. (S5) into Eq. (S2), the local concentration of H_2O_2 at a specific time (t) on the array is:

$$[H_2O_2]_t = \frac{k_r(1 - I(t)/I_0) - \mathrm{d}(I(t)/I_0)/\mathrm{d}t}{k_f \times I(t)/I_0} \quad \text{(S6)}$$

At the initial stage of the absorption reaction, we can assume that [SWNT] and [H_2O_2] are close to their original concentrations, allowing the forward reaction to completely dominate. Thus, Eq. (S3) can be simplified to:

$$\left.\frac{\mathrm{d}[H_2O_2-SWNT]}{\mathrm{d}t}\right|_{t\to 0} = k_f[H_2O_2]_0[SWNT]_0 \quad \text{(S7)}$$

By applying Eq. (S5) to the above equation, we can calculate the forward reaction constant (k_f) from the initial change in sensor intensity upon the addition of H_2O_2 in vitro:

$$k_f = \left.\frac{1}{[H_2O_2]_0}\frac{d(I(t)/I_0)dt}{dt}\right|_{t\to 0} \quad \text{(S8)}$$

By applying the data from Supplementary Fig. 11 to Eq. (S8), $k_f = 0.178\ M^{-1}sec^{-1}$. The value of k_r was determined to be 2.6 μsec^{-1}, based on the ratio between k_f and k_r calculated from the equilibrium dissociation constant K_D =14.7 μM[40].

Supplementary Note S2. Quantification of the H_2O_2 efflux at a single keratinocyte.

We calculated the actual amount of H_2O_2 efflux by a single keratinocyte using the localized H_2O_2 concentration on the SNI array. Assuming that each keratinocyte's efflux rate is the same, we derived the following relationship:

$$[\mathrm{H_2O_2}] \propto \text{Cell number} \tag{S9}$$

Thus, the degree of SNI's response (I/I_0), to H_2O_2 adsorption is proportional to the cell number (n) on the SNI. Each sample's time-series sensor response is normalized as:

$$\text{Normalized } I/I_0 = \frac{(n_1 \times \frac{I}{I_0} + n_2 \times \frac{I}{I_0} + n_3 \times \frac{I}{I_0})}{n_1+n_2+n_3} \tag{S10}$$

The cell number average ($n_{average}$) for each condition is expressed as $(n_1+n_2+n_3)/3$, and when we apply this to Supplementary Note Eq. (S6) for normalized I/I_0, we can express the efflux rate for each single keratinocyte's H_2O_2 output as:

$$\frac{[H_2O_2]_t}{n_{average}} = \frac{k_r(1-Normalized\ I(t)/I_0) - \mathrm{d}(Normalized\ I(t)/I_0)/\mathrm{d}t}{n_{average} \times k_f \times Normalized\ I(t)/I_0} \tag{S11}$$

Then, the area occupied by a single keratinocyte (A_C) on the SNI surface (A_S) is determined as:

$$A_C = A_S/\ n_{average} \tag{S12}$$

Assuming the adhered cell is shaped like the upper half of an ellipsoid, the volume of a single cell (V_C) is calculated as:

$$V_C = \frac{2}{3} \times A_C \times h \tag{S13}$$

Using the single-cell volume and the single efflux rate, the max value $[H_2O_2]_{max}$ from the time-series data is used to estimate the total H_2O_2 efflux under each UVA condition. The amount of single-cell H_2O_2 efflux is given by: Amount of single cell effluxed $\mathrm{H_2O_2} = [H_2O_2]_{max} \times V_C$

Supplementary Note S3. 3D Modeling of Time-Dependent H_2O_2 Efflux According to Directionality Considering Cell Morphology

Assuming the *xy*-plane as the reference plane for the cell and defining the cell's elliptical shape using its major axis (*a*) and minor axis (*b*), the time (*t*) dependent H_2O_2 efflux regions influenced by the cell's morphology can be expressed as:

$$\frac{x^2}{(a/2)^2} + \frac{y^2}{(b/2)^2} = t^2 \quad \text{(S14)}$$

By introducing a polar coordinate system with 16 equal angular divisions (∅), this can be rewritten as:

$$x = t\cos(\emptyset) \cdot \frac{\mathrm{a}}{\mathrm{b}},\ y = t\,sin(\emptyset) \quad \text{(S15)}$$

where:

$$\phi \in \left[\frac{(i-1)\cdot 2\pi}{16}, \frac{i\cdot 2\pi}{16}\right),\ i = 1,2,\ldots,16 \quad \text{(S16)}$$

This transformation allows the elliptical shape representing the cell to be converted from traditional *x*, *y* coordinates into direction- and time-dependent coordinates, maintaining the same time frame as the distance from the cell core increases. When the *z*-axis incorporates sensor responses fitted to Eq. (2):

$$z(\phi, t) = \frac{A_i}{t\sigma_i\sqrt{2\pi}} \exp\left(-\frac{(\ln t - \mu_i)^2}{2\sigma_i^2}\right) \quad \text{(S17)}$$

a 3D graph can be generated, enabling examination of the directional characteristics of H_2O_2 efflux from a single cell while considering its morphology.

A

	Sequence	Full sequence
C-SWNT	$(CCCT)_7$	CCCTCCCTCCCTCCCTCCCTCCCTCCCT

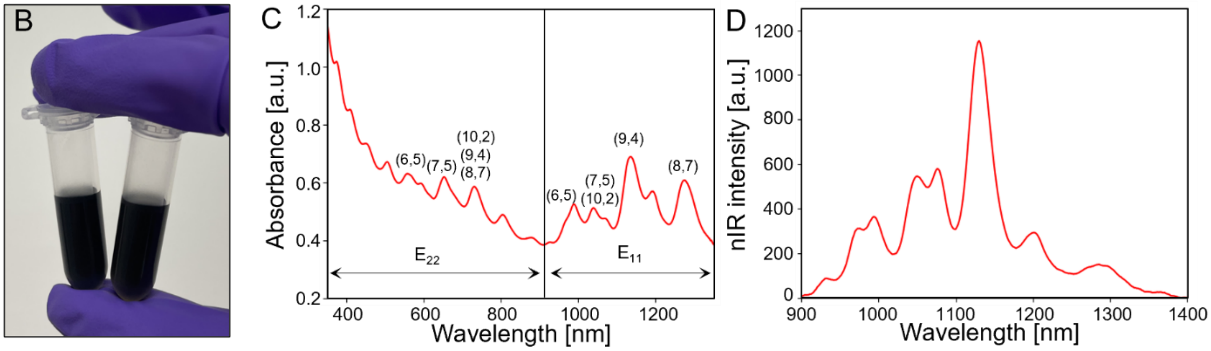

Fig. S1. Design and synthesis of DNA/SWNT for H_2O_2 sensing. (**A**) DNA sequence for corona interface formation on SWNTs. (**B**) Photograph, (**C**) UV-vis-nIR absorption spectra and (**D**) nIR fluorescence spectra of C-SWNTs.

A

Sequence	Full sequence
$(GT)_{15}$	GTGTGTGTGTGTGTGTGTGTGTGTGTGT
$(AT)_{15}$	ATATATATATATATATATATATATATATAT
$(CT)_{15}$	CTCTCTCTCTCTCTCTCTCTCTCTCTCTCT
$(CC)_{15}$	CCCCCCCCCCCCCCCCCCCCCCCCCCCC

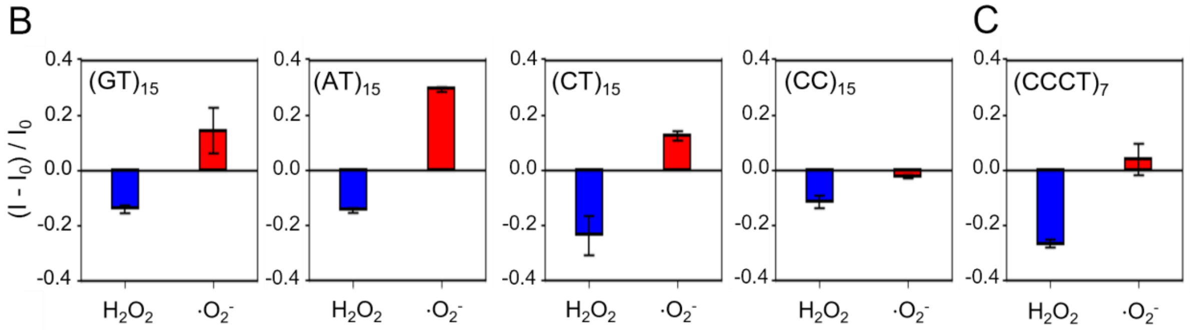

Fig. S2. ROS response comparison of candidate sequences for deriving $(CCCT)_7$. (A) Pair-repeated DNA sequence for corona interface formation on SWNTs. Bar graphs showing the cross-selectivity test results of **(B)** pair-repeated DNA-SWNTs and **(C)** C-SWNT between H_2O_2 and $\cdot O_2^-$ (100 µM). The results showed minimal reactivity to $\cdot O_2^-$ for sequences containing only cytosine (C), likely due to the single-ring structure of the pyrimidine base, while the cytosine-thymine (CT) combination demonstrated improved reactivity to H_2O_2 but insufficient selectivity against $\cdot O_2^-$. To enhance cross-selectivity, the sequence design was modified from pairwise base repeats to a tetranucleotide repeat pattern, resulting in the 28-mer sequence $(CCCT)_7$, which exhibited excellent sensitivity to H_2O_2 and high cross-selectivity against $\cdot O_2^-$.

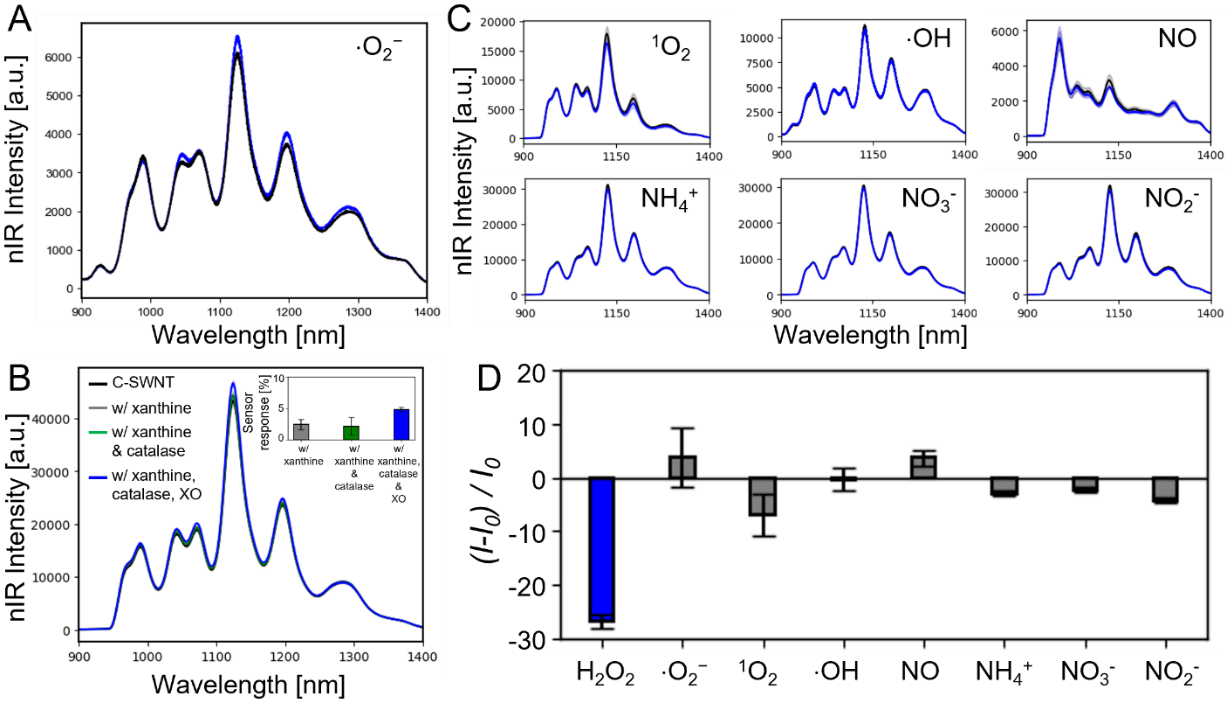

Fig. S3. H_2O_2 sensing performance of C-SWNT towards biologically relevant small molecules under buffered conditions. (**A**) nIR fluorescence spectra of C-SWNT in PBS (pH 7.4) before (black line) and after the generation of 100 µM superoxide ($\cdot O_2^-$, blue line). (**B**) Sequential response measurements upon the addition of xanthine (gray line), catalase (green line), and xanthine oxidase (blue line). (**C**) nIR fluorescence spectra of C-SWNT before (black line) and after exposure to 100 µM of various analytes, including $\cdot O_2^-$, singlet oxygen ($^1O_2$), hydroxyl radical ($\cdot OH$), nitric oxide (NO), ammonium (NH_4^+), nitrate (NO_3^-), and nitrite (NO_2^-) (blue line). The mean values are shown as solid lines, with shaded areas representing the standard deviation (σ) across $n = 3$ technical replicates. (**D**) Calculated nIR sensor responses of C-SWNT for each analyte.

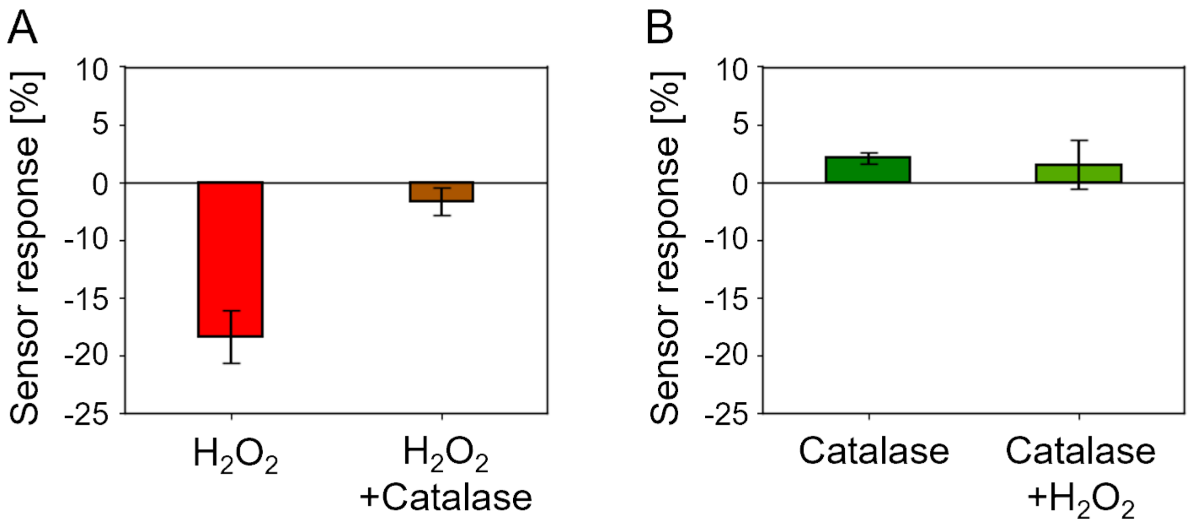

Fig. S4. Reversibility test of L-SWNT toward H_2O_2 using catalase. (**A**) Bar graph of the quenching response of L-SWNT to 50 μM H_2O_2 and its recovery by 500 U/mL catalase. (**B**) Bar graph showing the response of L-SWNT to catalase followed by H_2O_2 in sequence.

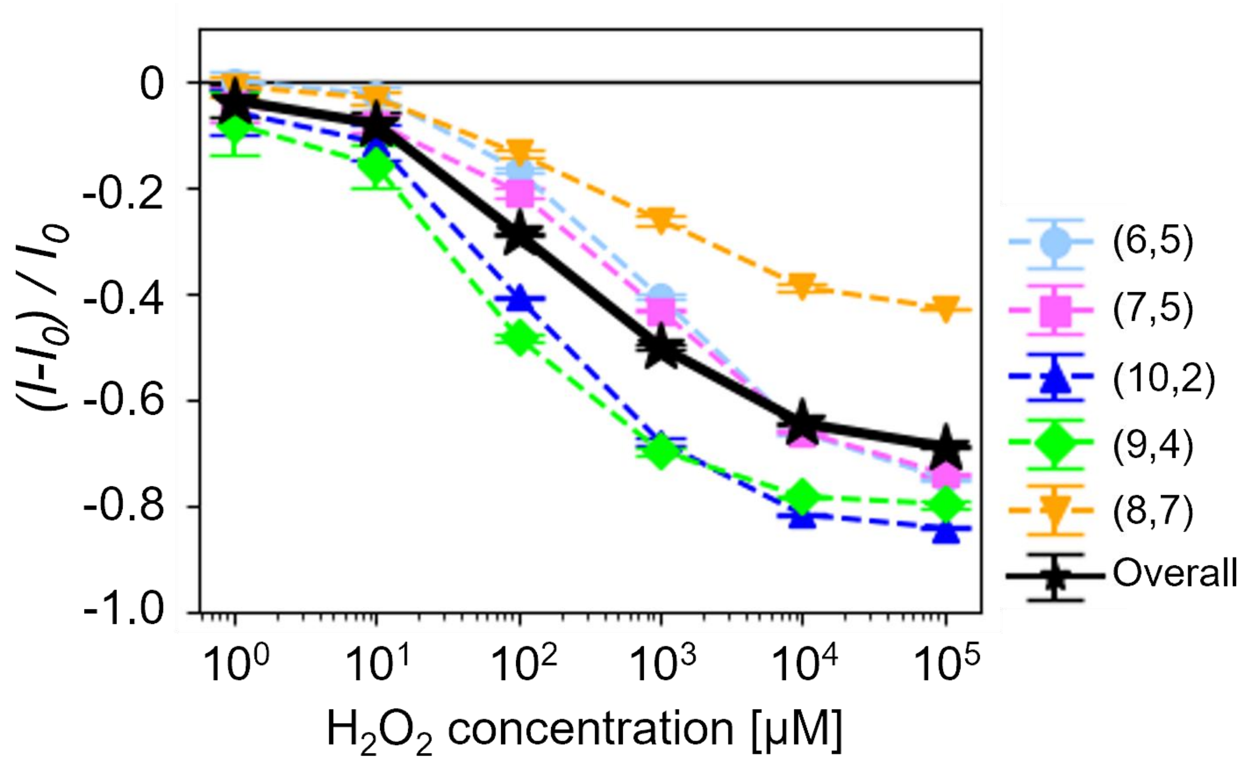

Fig. S5. Peak intensity changes of each chirality of L-SWNT in response to varying concentrations (1 to 10^5 μM) of H_2O_2.

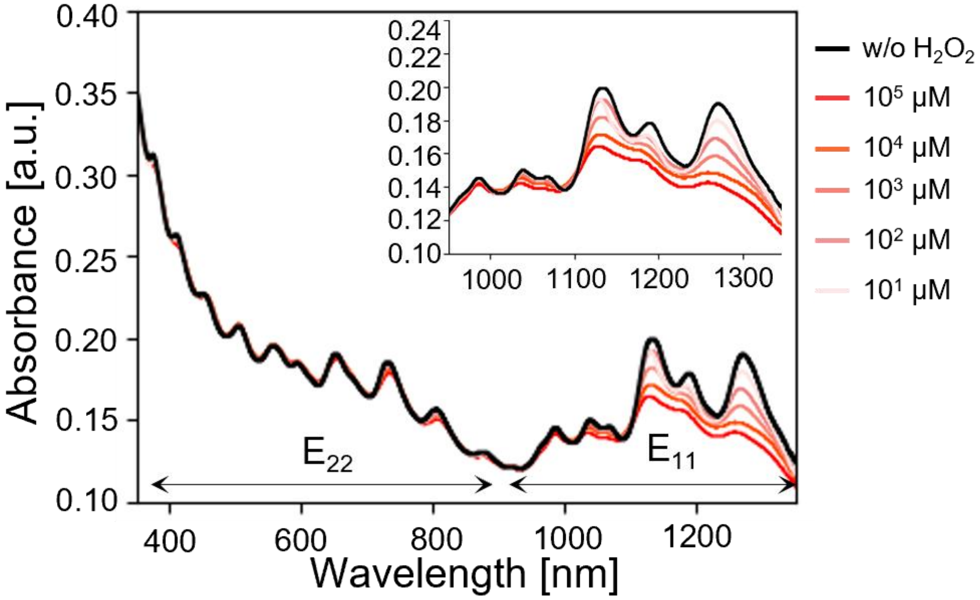

Fig. S6. UV-vis-nIR absorption response spectra of L-SWNT at various concentrations of H_2O_2. Definition of the 350–950 nm range as the E_{22} transition region and the 950–1350 nm range as the E_{11} transition region, with an enlarged graph showing spectral changes in the E_{11} region.

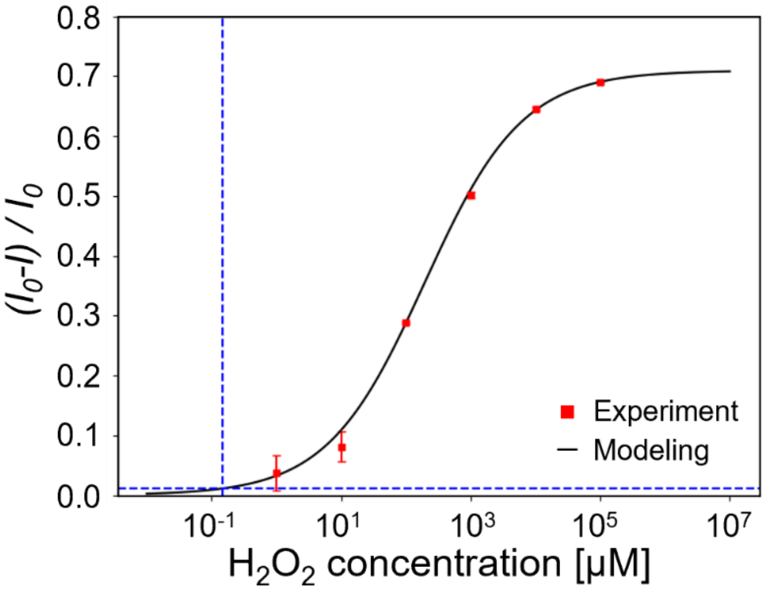

Fig. S7. Calibration curve of L-SWNT to H_2O_2. A calibration curve of L-SWNT for H_2O_2. Fitting the data from Fig. 2A to Eq. (1) yielded an R^2 value of 0.998, with a proportionality factor (α) of 0.71, an equilibrium dissociation constant (K_D) of 20.9 μM, and cooperativity of the binding reaction of 0.58. The intersection of the blue dashed lines represents the limit of detection (LOD), and this value was calculated by defining the nanosensor response in water as the noise level (σ) and using a signal corresponding to three times this noise level.

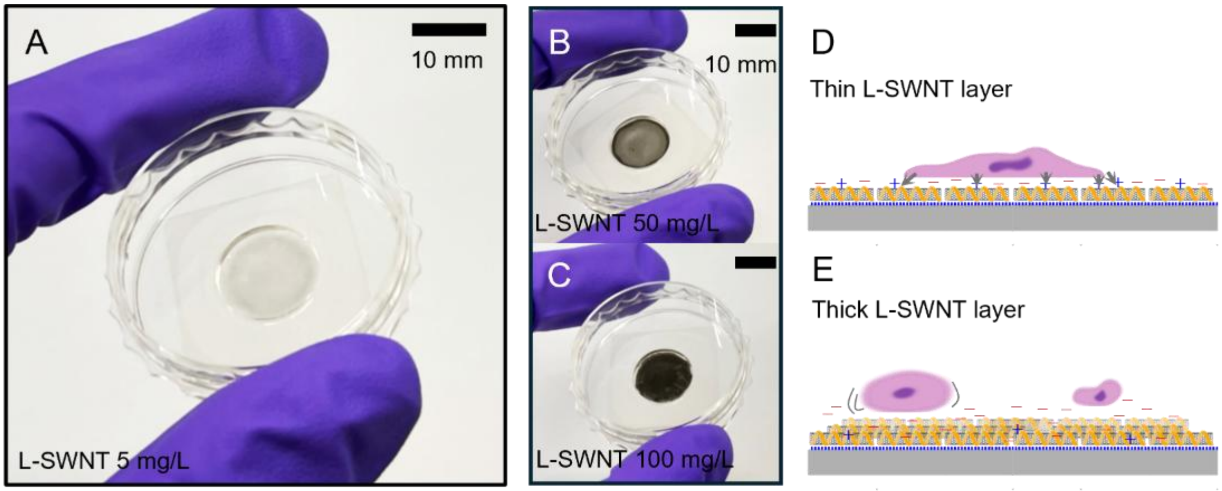

Fig. S8. Photographs and schematic illustrations of SNI fabricated with various L-SWNT concentrations. Photographs of the coated L-SWNTs array on the glass substrates with (**A**) 5 mg/L, (**B**) 50 mg/L, and (**C**) 100 mg/L. Schematic illustrations of skin cell adhesion behavior on (**D**) a thin (*e.g.* 5 mg/L) and (**E**) a thick (*e.g.* 50, 100 mg/L) L-SWNT layer.

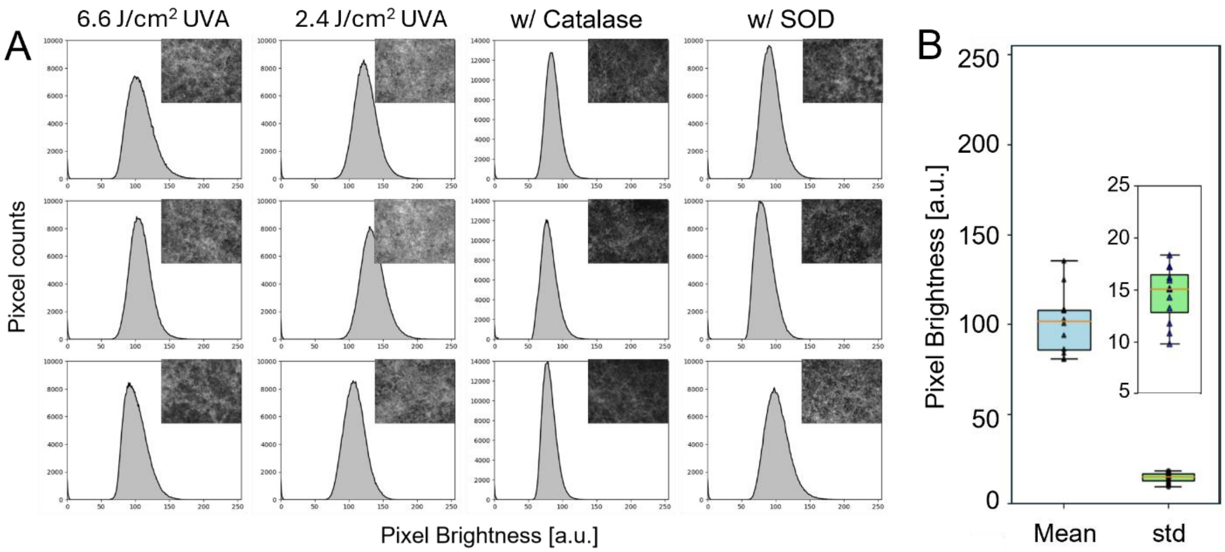

Fig. S9. Quantification of spatial distribution of SNI's nIR fluorescence, used in further condition-specific photoaging monitoring. (**A**) Raw nIR fluorescence images and pixel brightness distributions of SNI under 6.6 J/cm^2 UVA (leftmost), 2.4 J/cm^2 UVA (second from left), w/ Catalase (second from right), and w/ SOD (rightmost) photoaging monitoring conditions. (**B**) Box plot of the mean brightness (101.14 ± 16.56) across the full area and the standard deviation (14.57 ± 2.58) of each image.

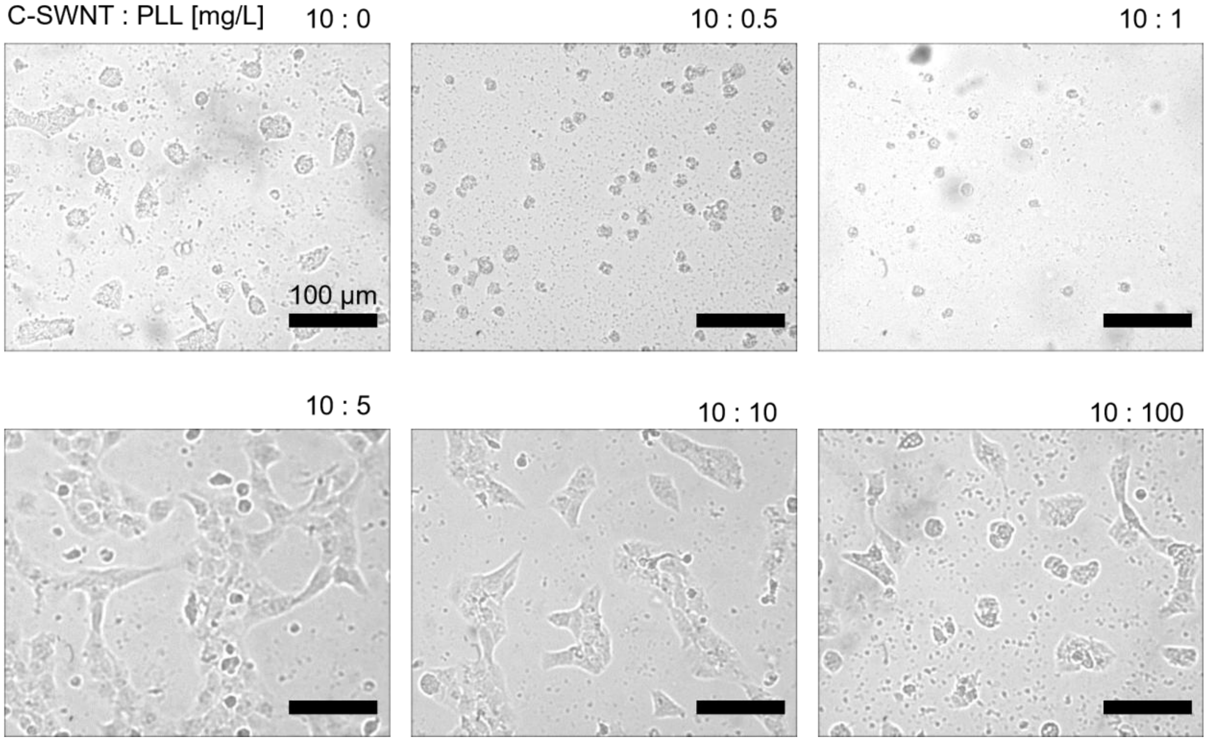

Fig. S10. Optical microscope images of cell proliferation on SNI. Bright-field images of keratinocytes seeded on SNI fabricated with L-SWNTs at various PLL concentrations. Images were taken after 24 hr post-seeding.

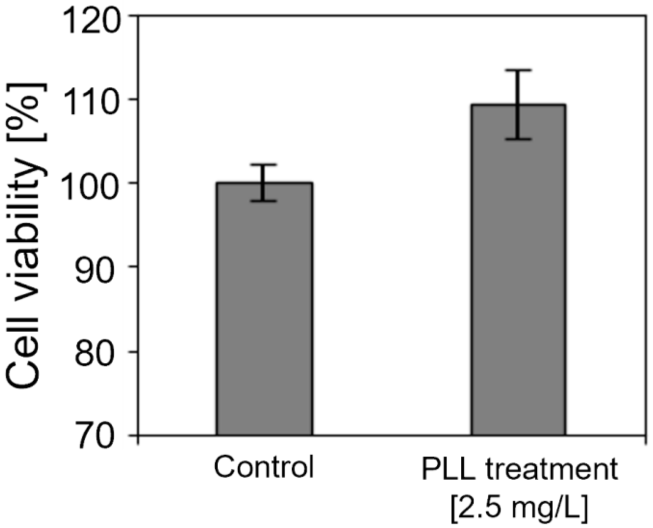

Fig. S11. **Cell viability measurement based on PLL treatment.** The data represent the mean value of n = 3 biological replicates.

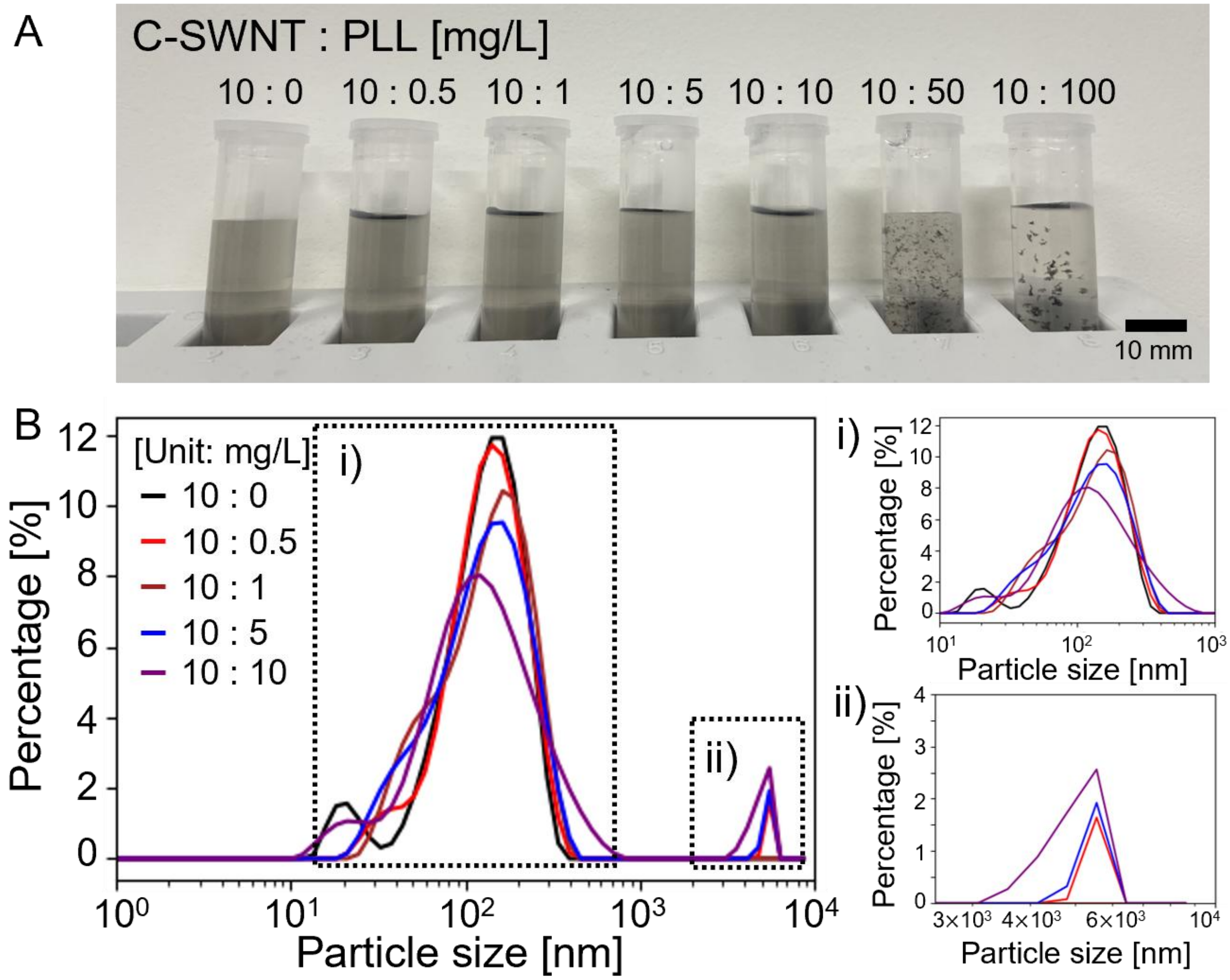

Fig. S12. Photograph and particle size measurement of L-SWNT. (**A**) Photograph of C-SWNTs mixed with various PLL concentrations after 6 hr. (**B**) DLS data of L-SWNTs, excluding the concentrations where visible aggregation was observed (10:50, 10:100). i) Enlarged graph for the 100 nm range. ii) Enlarged graph for the 1–10 µm range**.**

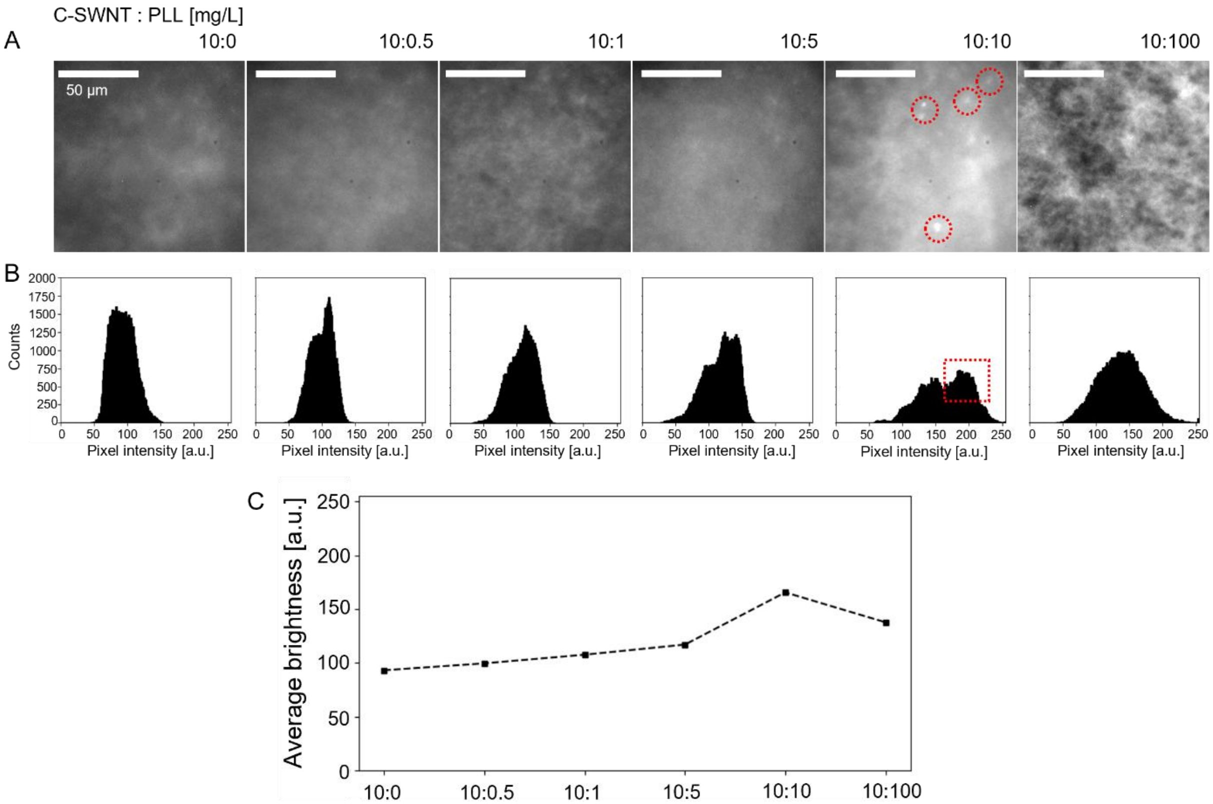

Fig. S13. Coating of L-SWNTs at various concentrations of PLL and pixel brightness distribution. (**A**) nIR images of L-SWNT coatings on glass substrates with various PLL concentrations. (**B**) Distribution of pixel intensity. The red dotted line indicates the region and distribution of aggregated L-SWNTs. (**C**) Average nIR pixel intensity across the entire coated area.

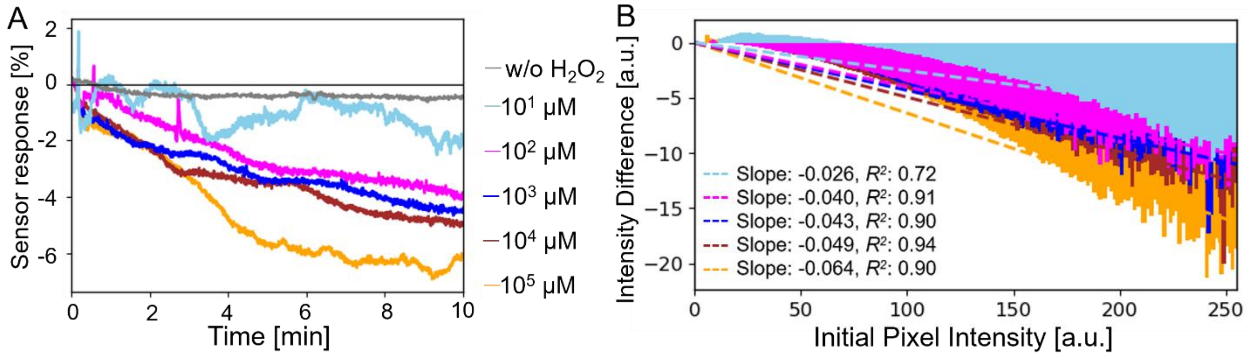

Fig. S14. Real-time nIR response of the nanosensor interface to various concentrations of H_2O_2. (**A**) Time-series data for each H_2O_2 concentration. The data represent the mean values of $n = 3$ replicates. (**B**) Changes in brightness for individual pixels relative to their original brightness at the endpoint of the nIR response to various H_2O_2 concentrations. The data represent the mean values of $n = 3$ replicates.

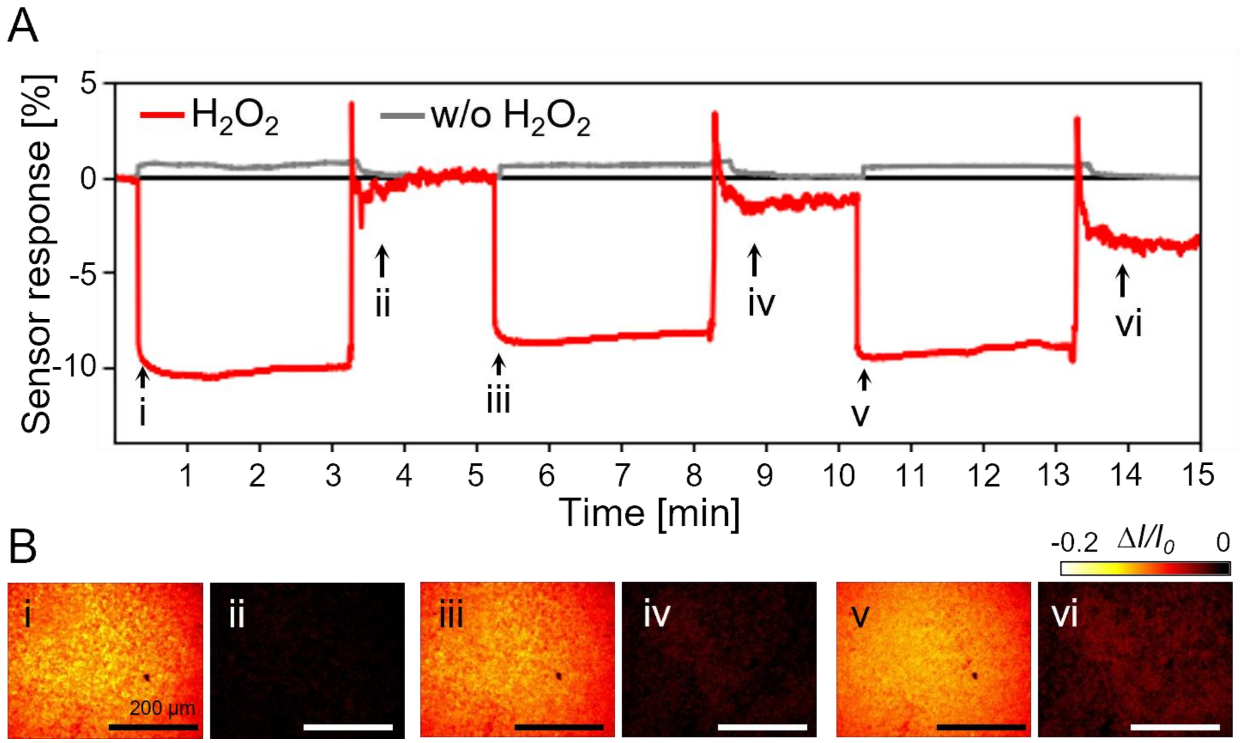

Fig. S15. Reversibility test of SNI toward H_2O_2. (A) Real-time sensor responses during three drop-and-retrieve cycles of H_2O_2 (red) and control without H_2O_2 (gray). Arrows indicate specific timepoints during the cycles. **(B)** nIR sensor response images corresponding to the timepoints (i, ii, iii, iv, v, vi) labeled in (A). Residual H_2O_2 from the pipette retrieval process was observed but did not affect the sensor's reactivity.

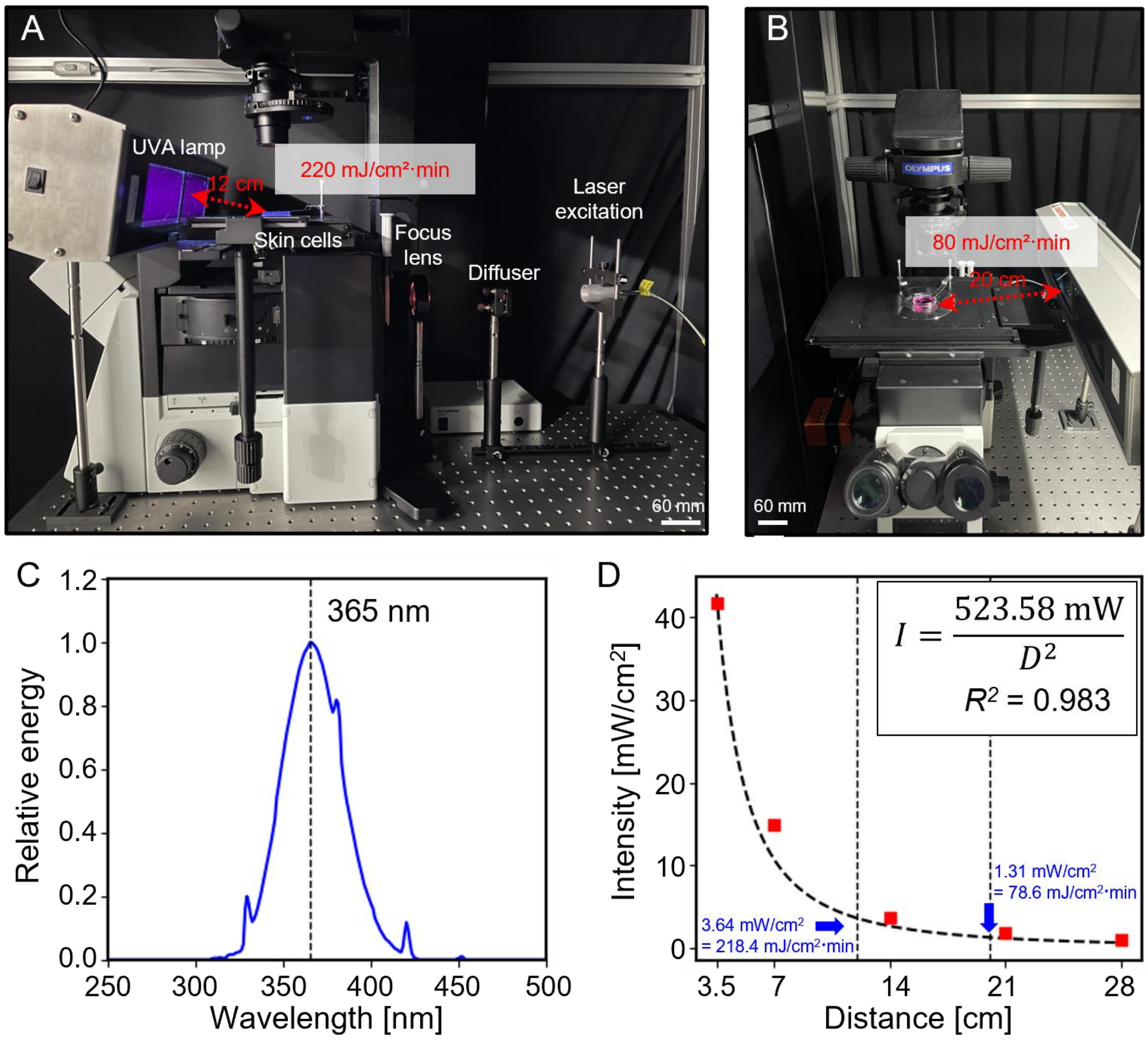

Fig. S16. Photographs and characterization of the photoaging monitoring setup under daily UVA exposure. Distance control of the lamp for (**A**) 6.6 J/cm^2 UVA exposure, and (**B**) 2.4 J/cm^2 UVA exposure. (**C**) Irradiance spectrum profile of the UVA lamp (purchased from Vilber Bio Imaging). (**D**) Measured UVA intensity (*I*) as a function of distance (*D*) using a power meter (PM100D, Thorlabs), demonstrating a strong inverse square relationship ($R^2 = 0.983$). Irradiance was estimated as 3.64 mW/cm^2 at 12 cm and 1.31 mW/cm^2 at 20 cm, consistent with reported UVA levels on clear (~2 mW/cm^2) and overcast (~0.8 mW/cm^2) days (*89*).

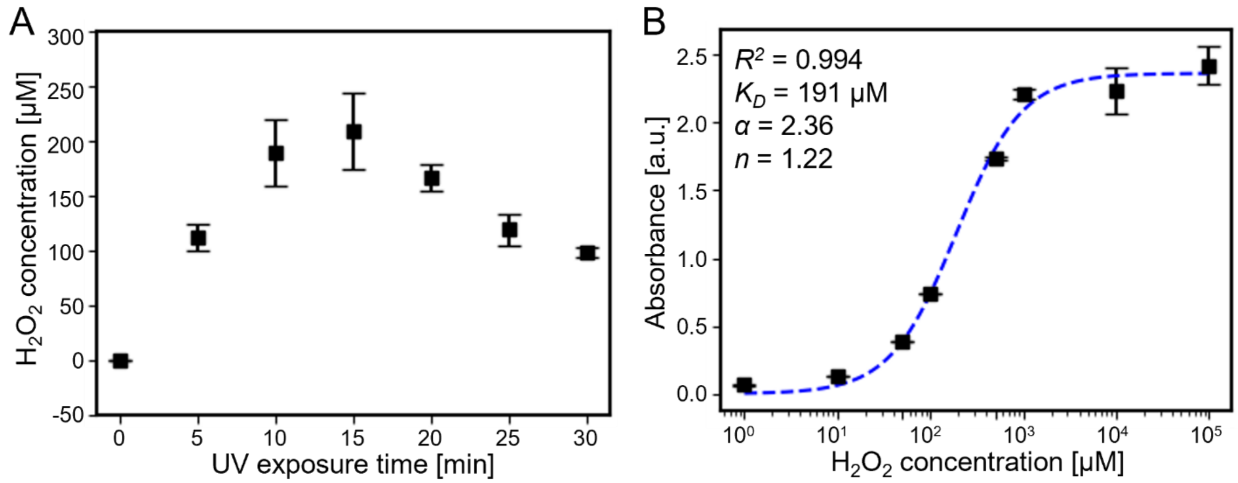

Fig. S17. Results from the photometric H_2O_2 assay kit (118789, Merck Millipore). (**A**) H_2O_2 levels measured every 5 minutes under high UVA conditions, showing a peak concentration of 208 µM at 15 minutes. The data points represent the mean, and the error bars indicate σ from n = 5 independent biological replicates. (**B**) Calibration curve of the assay kit response as a function of H_2O_2 concentration. The response was fitted using Eq. (1), and the detection range is within the µM scale. The data points represent the mean, and the error bars indicate σ from n = 3 replicates.

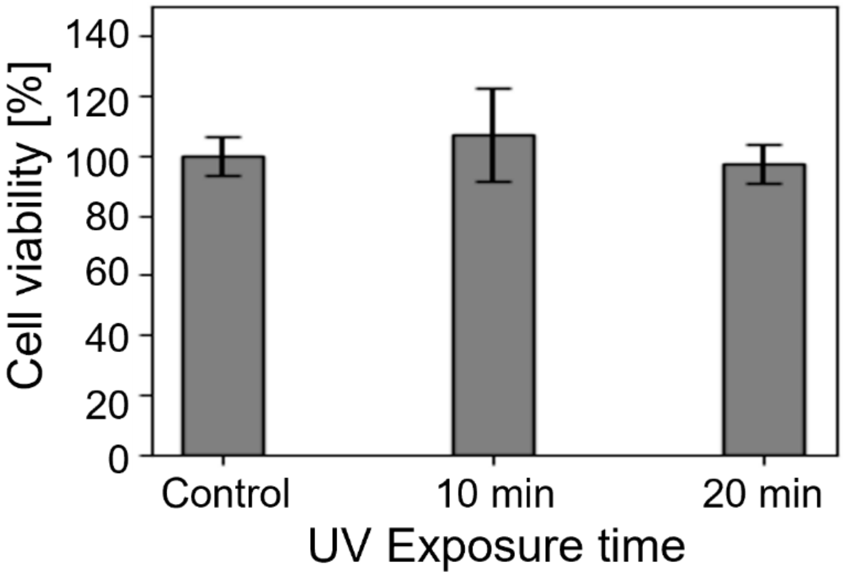

Fig. S18. Cell viability measurement of the daily UVA exposure photoaging setup. Viabilities of keratinocytes grown on SNI under different UVA exposure times (0, 10, and 20 min), as measured by colorimetric cell viability assay kit in the daily UVA exposure photoaging setup.

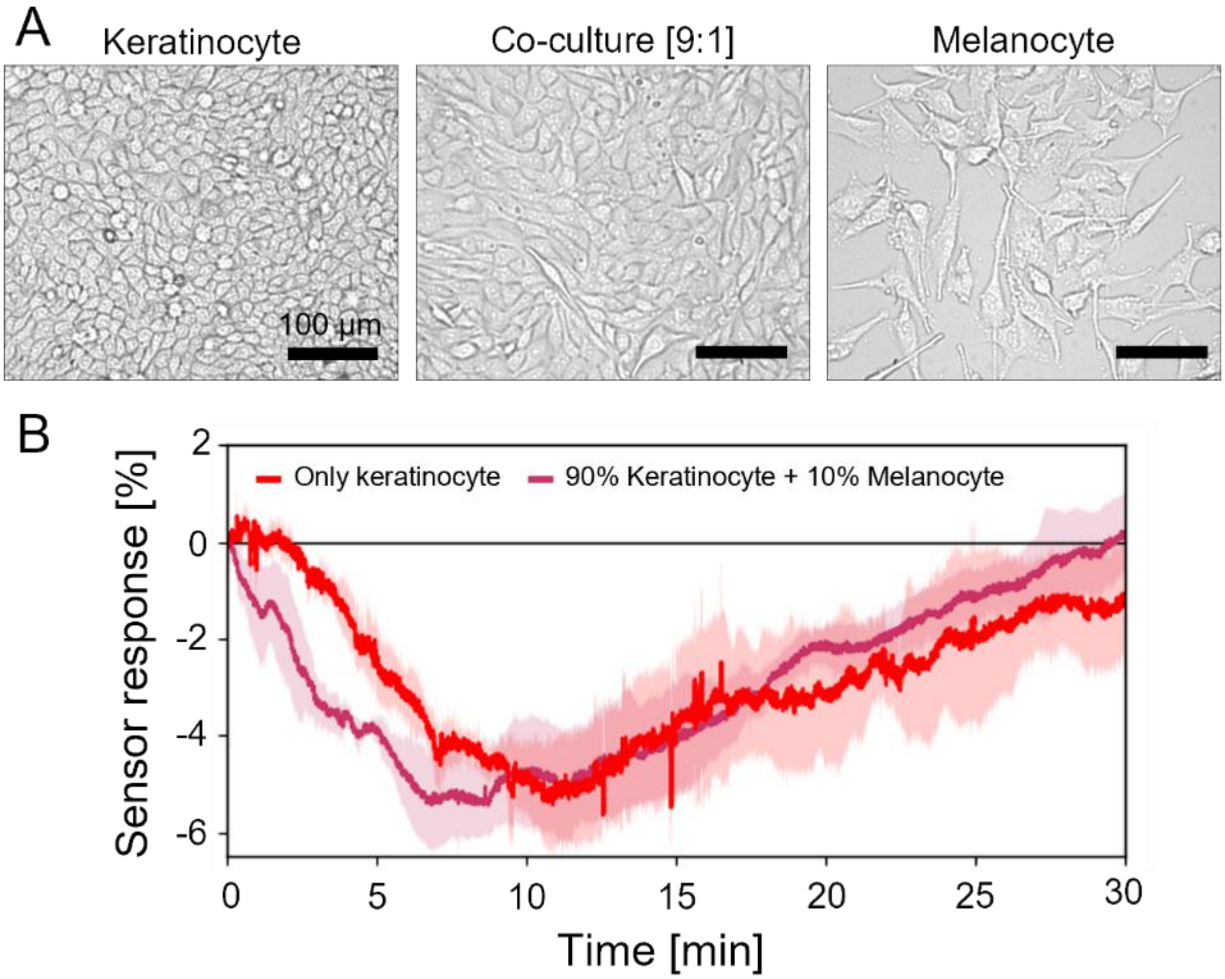

Fig. S19. Real-time monitoring of photoaging in keratinocyte and melanocyte coculture using SNI. (**A**) Brightfield images of cells cultured on SNI, including only keratinocytes (left), keratinocyte and melanocyte coculture (9:1, middle), and only melanocytes (right). Fig. S18A-left is identical to Fig. 2E (48 hr) as both originate from the same experiment. (**B**) Real-time sensor responses of the nanosensor array beneath keratinocyte and melanocyte cocultured samples. The solid line represents the mean, and the shaded region indicates σ from n = 3 biological replicates.

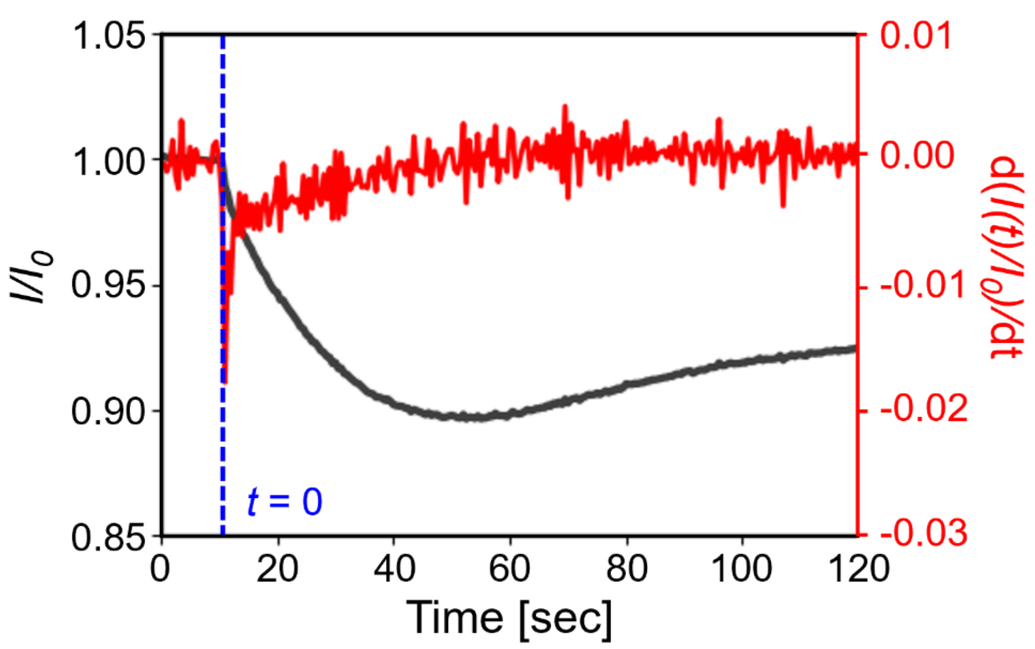

Fig. S20. Differentiation graph of the SNI response to 100 mM H_2O_2. Differentiation graph (red line) of the SNI response to 100 mM H_2O_2, obtained by dividing the time intervals at each data point of Fig. 2I (black line). The blue vertical line represents the point where $t = 0$, marking the moment when the forward reaction becomes dominant ($y = -0.0178$).

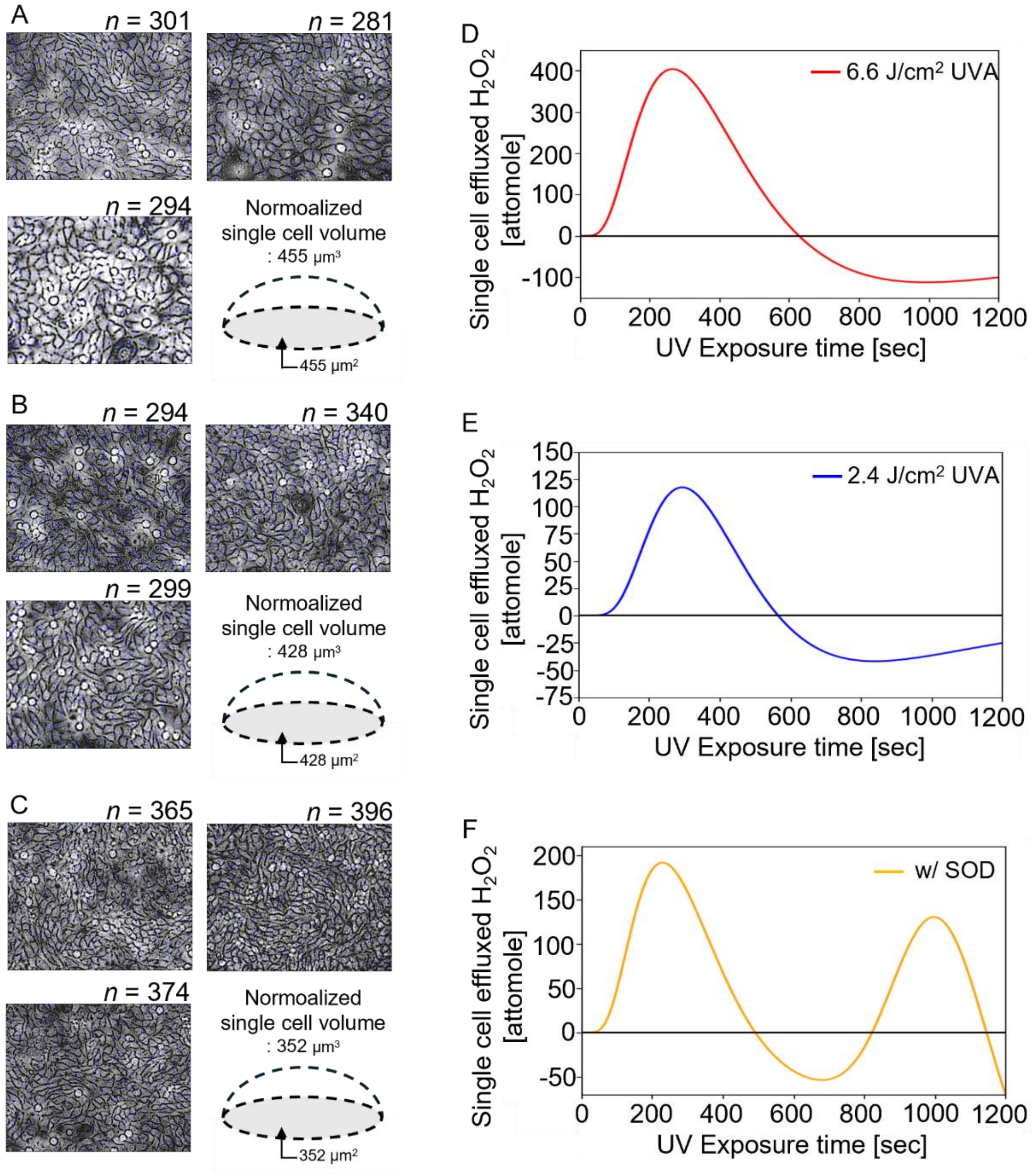

Fig. S21. Cell counting and time-series data adjusted for cell numbers. The cell numbers for each sample were determined using bright-field images of each sample, with Fiji's "Subtract Background" function and cell counting plugin. (**A**) Under 6.6 J/cm^2 UVA exposure. (**B**) Under 2.4 J/cm^2 UVA exposure. (**C**) Under 6.6 J/cm^2 UVA exposure with SOD treatment. (**D**), (**E**), (**F**) correspond to time-series data of single-cell H_2O_2 efflux for each respective condition.

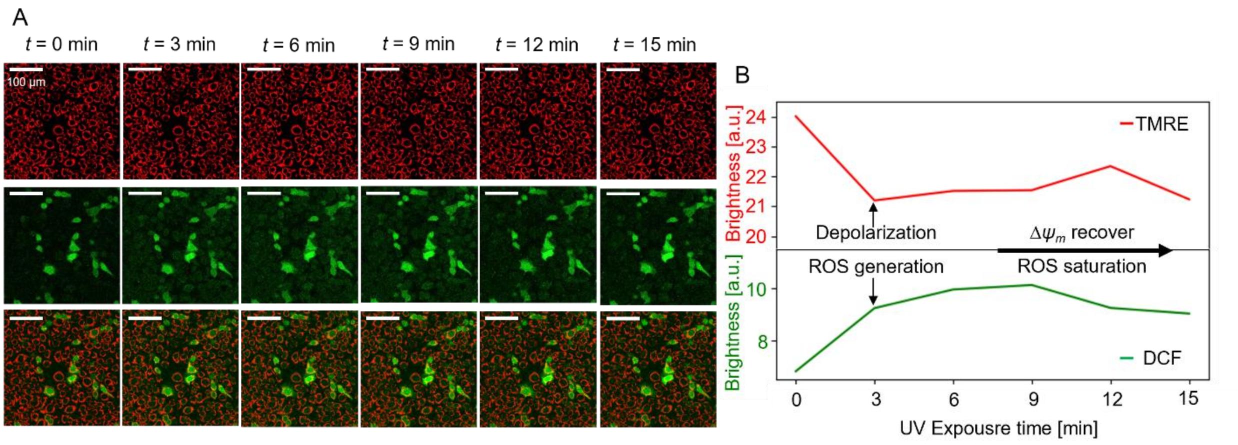

Fig. S22. Detection of mitochondrial membrane depolarization using TMRE and intracellular ROS accumulation using H_2DCFDA under 6.6 J/cm^2 UVA exposure. (**A**) Confocal images taken with 3 min intervals of TMRE (top), H_2DCFDA (middle), and merged images (bottom). (**B**) Average brightness of images (TMRE: red channel, H_2DCFDA: green channel) measured at each time interval using Fiji.

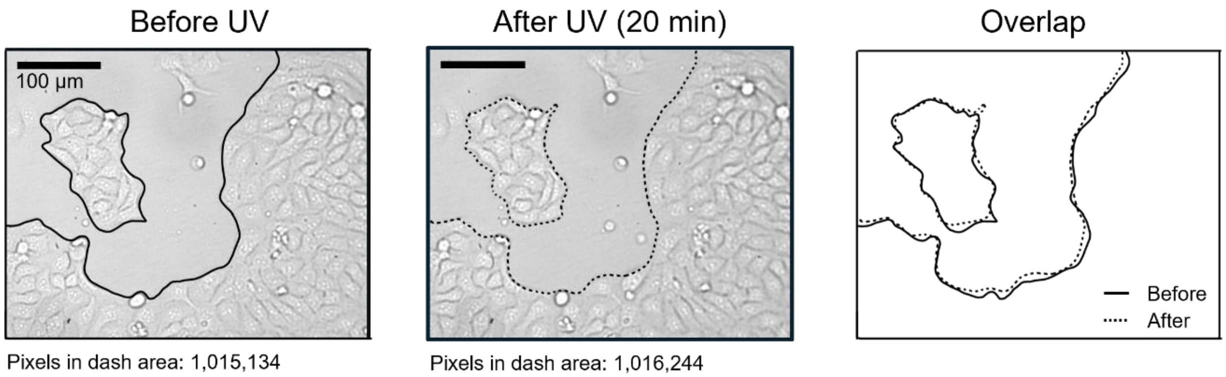

Fig. S23. Cell location changes before and after 20 min of 6.6 J/cm^2 UVA exposure. There was a 0.109% increase in the area after 20 min of 6.6 J/cm^2 UVA exposure, compared to the area before exposure.

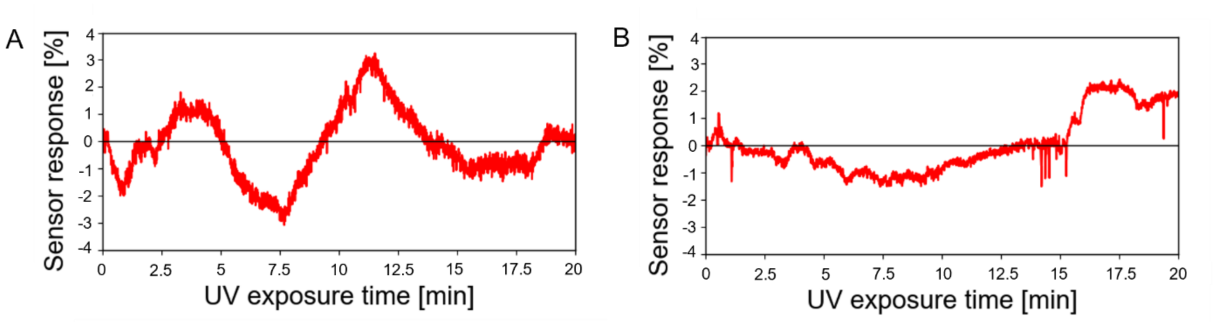

Fig. S24. Time-series nIR sensor response data under 6.6 J/cm^2 UVA exposure for controlled cell culture conditions. (**A**) nIR sensor response from the top of Fig. 4C. (**B**) nIR sensor response from the bottom of Fig. 4C**.**

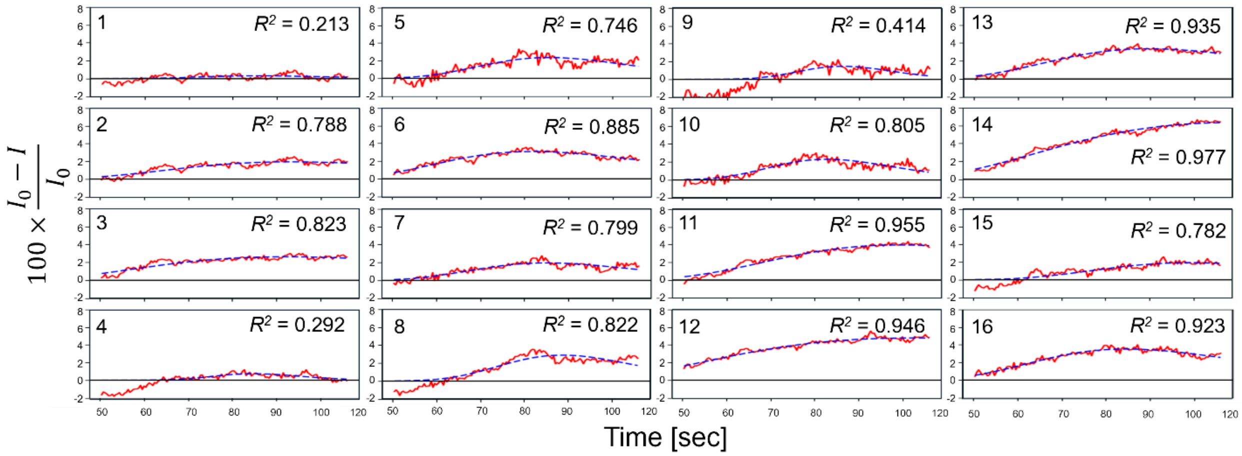

Fig S25. Raw real-time sensor responses at high magnification (100X) photoaging screening. Individual sensor responses from each of the 16 divided regions in Fig. 4J are fitted to Eq. (2) and presented as separate graphs.

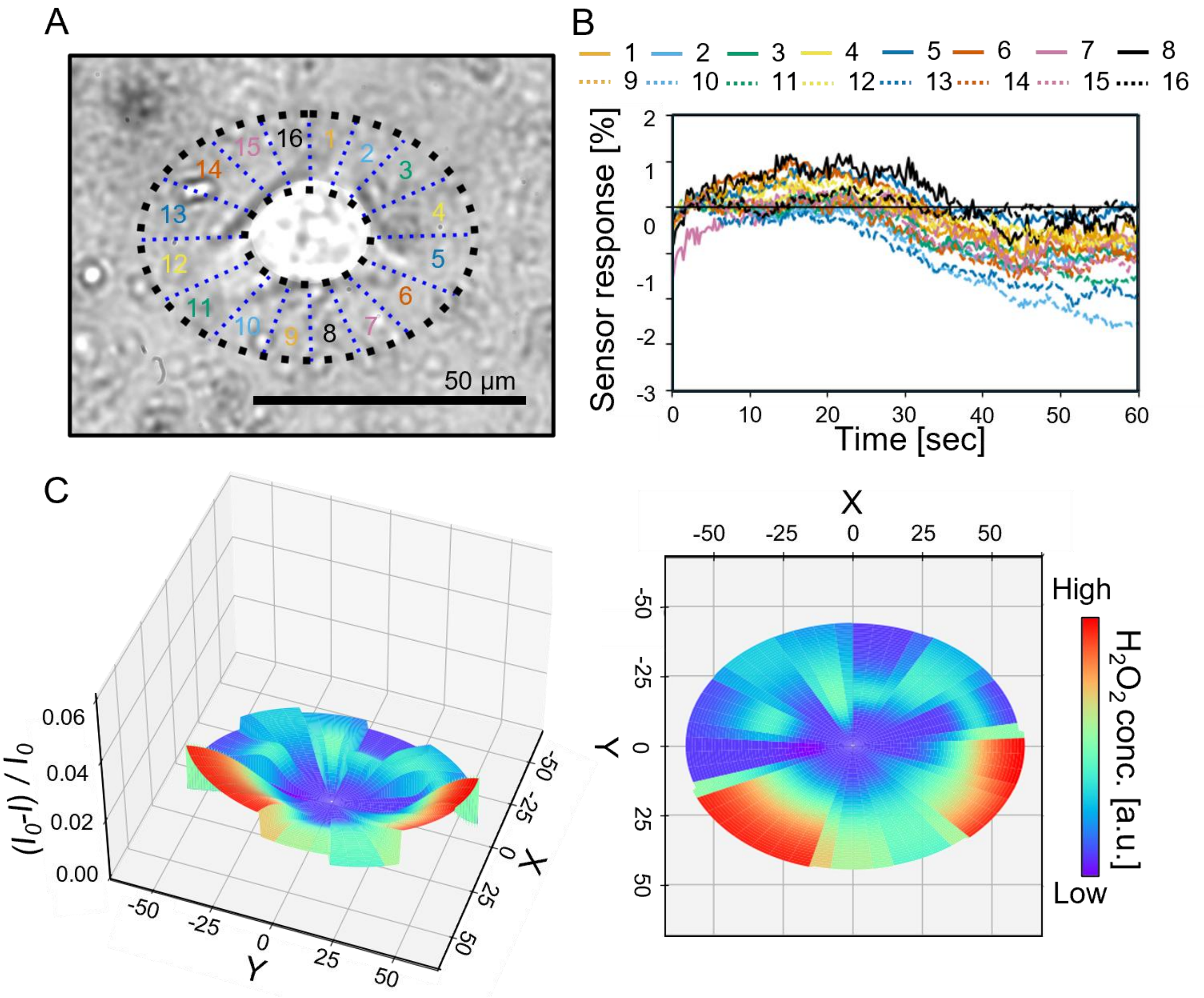

Fig S26. **Spatiotemporal analysis of a single keratinocyte's H_2O_2 efflux.** (**A**) Bright field image of a designated elliptical region divided into 16 equal sections by radial lines (blue) originating from the cell core. (**B**) Real-time sensor responses represented by the corresponding colors of the 16 defined sections. (**C**) 3D graphs of fitted time-series H_2O_2 efflux from a single keratinocyte with an elevated view (left) and a top view (right).

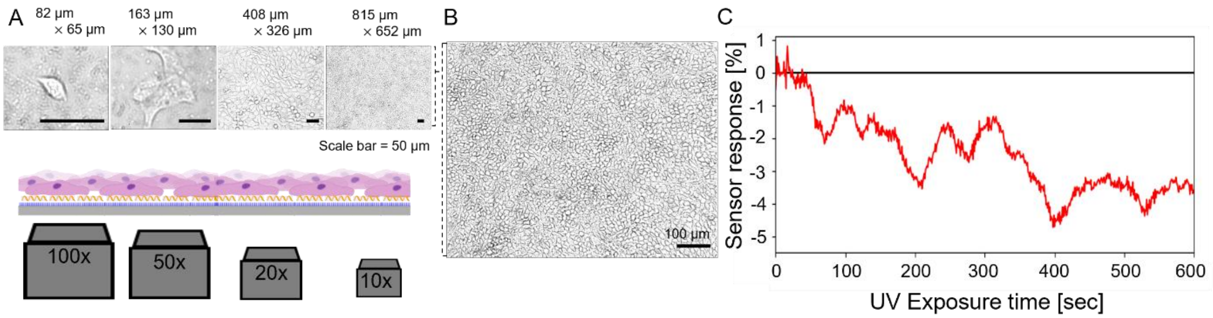

Fig. S27. Time-series sensor response data at low magnification. (**A**) Brightfield images of keratinocytes on SNI at different microscope magnifications (100X, 50X, 20X, and 10X). (**B**) Brightfield image of keratinocytes on SNI at 10X magnification. (**C**) Time-series nIR sensor response data under 6.6 J/cm^2 UVA exposure at 10X magnification.

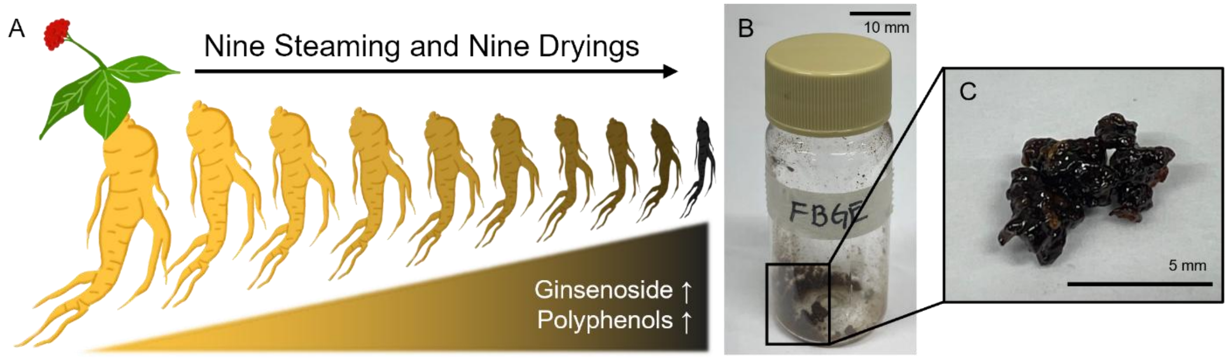

Fig. S28. Preparing process of Fermented Black Ginseng Extract (FBGE). (**A**) Schematic illustrations of the nine steaming and nine drying cycles. Through this process, the ginseng is transformed into black ginseng with higher ginsenoside and polyphenol content, followed by fermentation with *Bacillus megaterium*. (**B**) Photograph and (**C**) magnified image of FBGE powders.

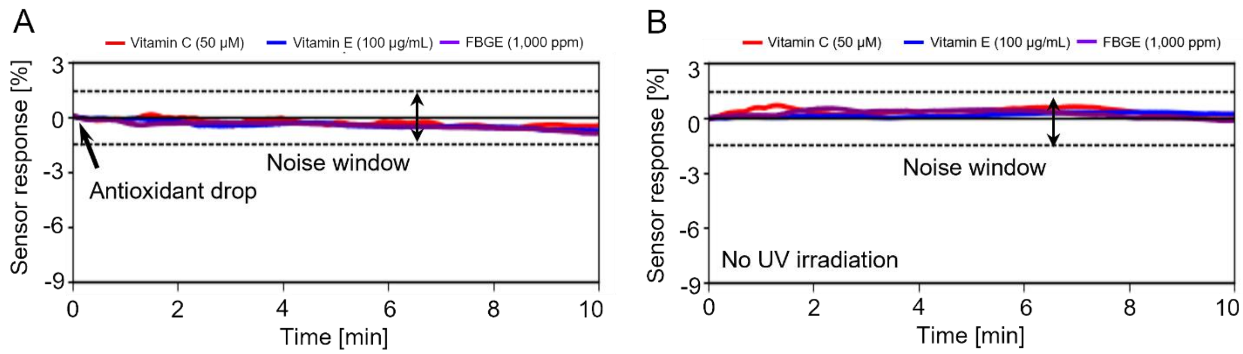

Fig. S29. Real-time nIR response of the SNI during keratinocyte culturing with antioxidant treatment. Time-series nIR responses of the SNI **(A)** immediately after antioxidant treatment and **(C)** after 24 hours of incubation without UV irradiation. (Black dashed line: $|y|$ = 1.56 %, 3σ of **Fig. 2H**).

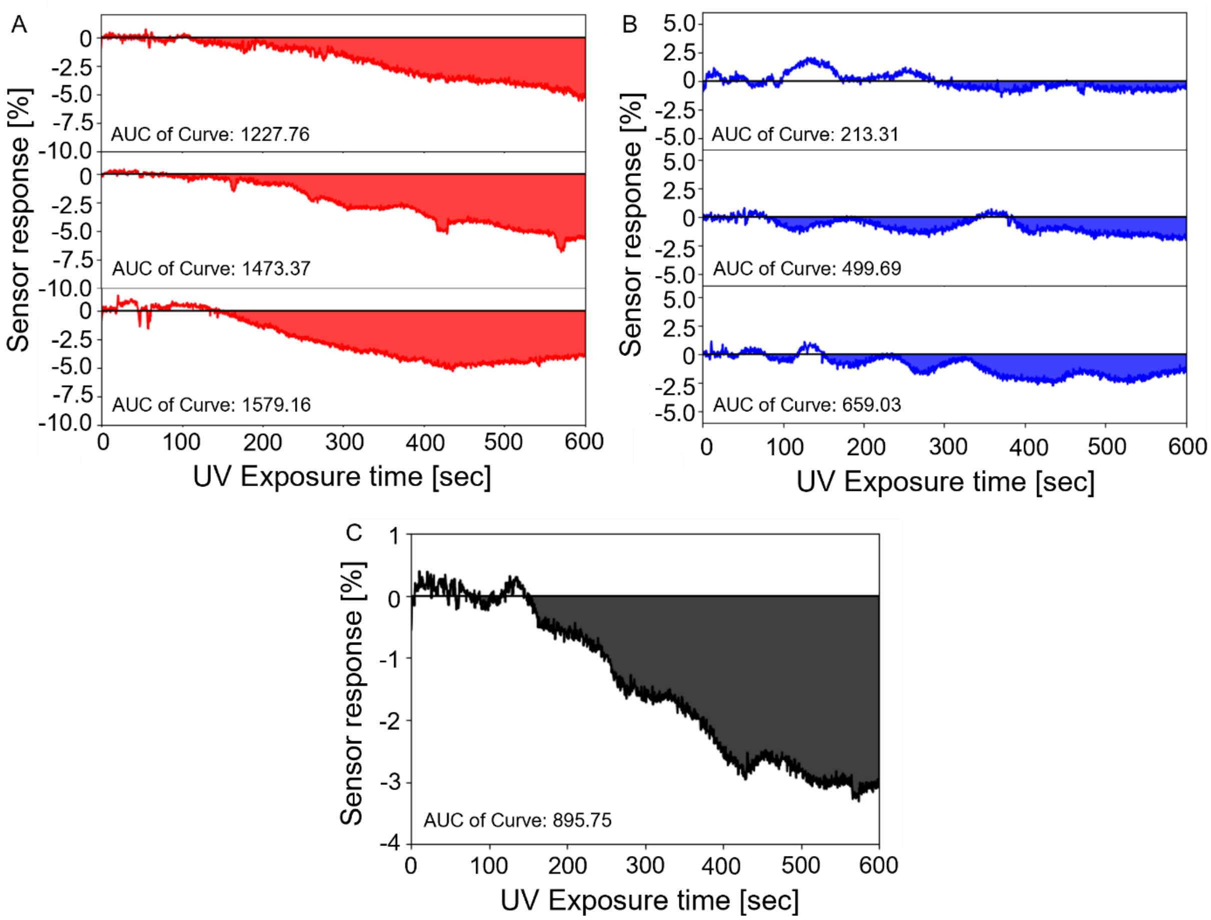

Fig. S30. Standard photoaging curve. (**A**) Time-series nIR sensor response data and AUC values for each sample under 6.6 J/cm^2 UVA exposure, shown in **Fig. 2B**. (**B**) Corresponding data and values under 2.4 J/cm^2 UVA exposure, shown in Fig. 2C. (**C**) Defined standard photoaging curve and associated AUC value.

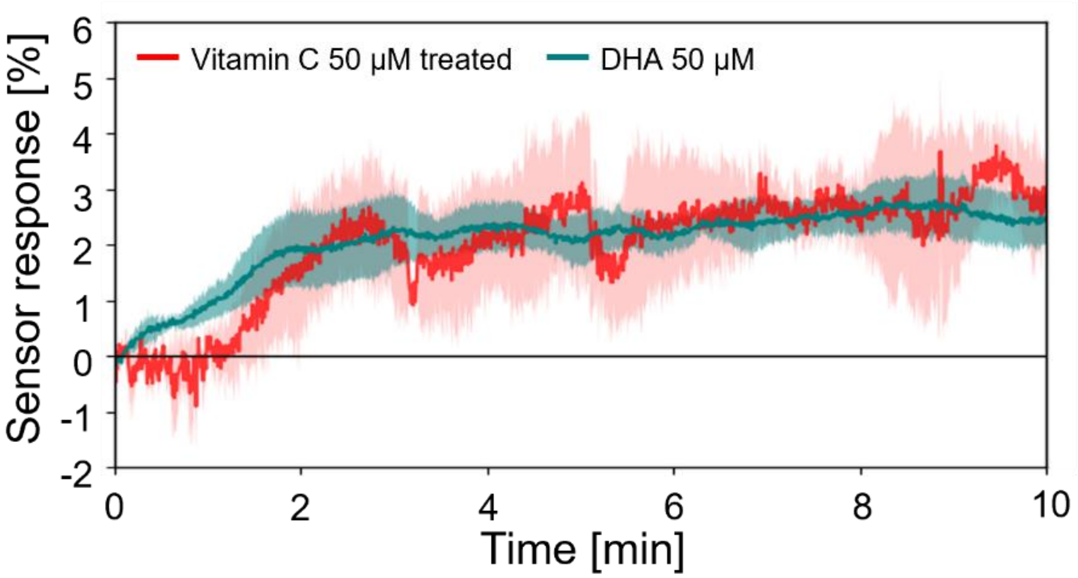

Fig. S31. Real-time nIR response of the nanosensor interface to 50 µM DHA. The solid line representing the mean and the shaded region indicating *σ* from *n* = 3 replicates.

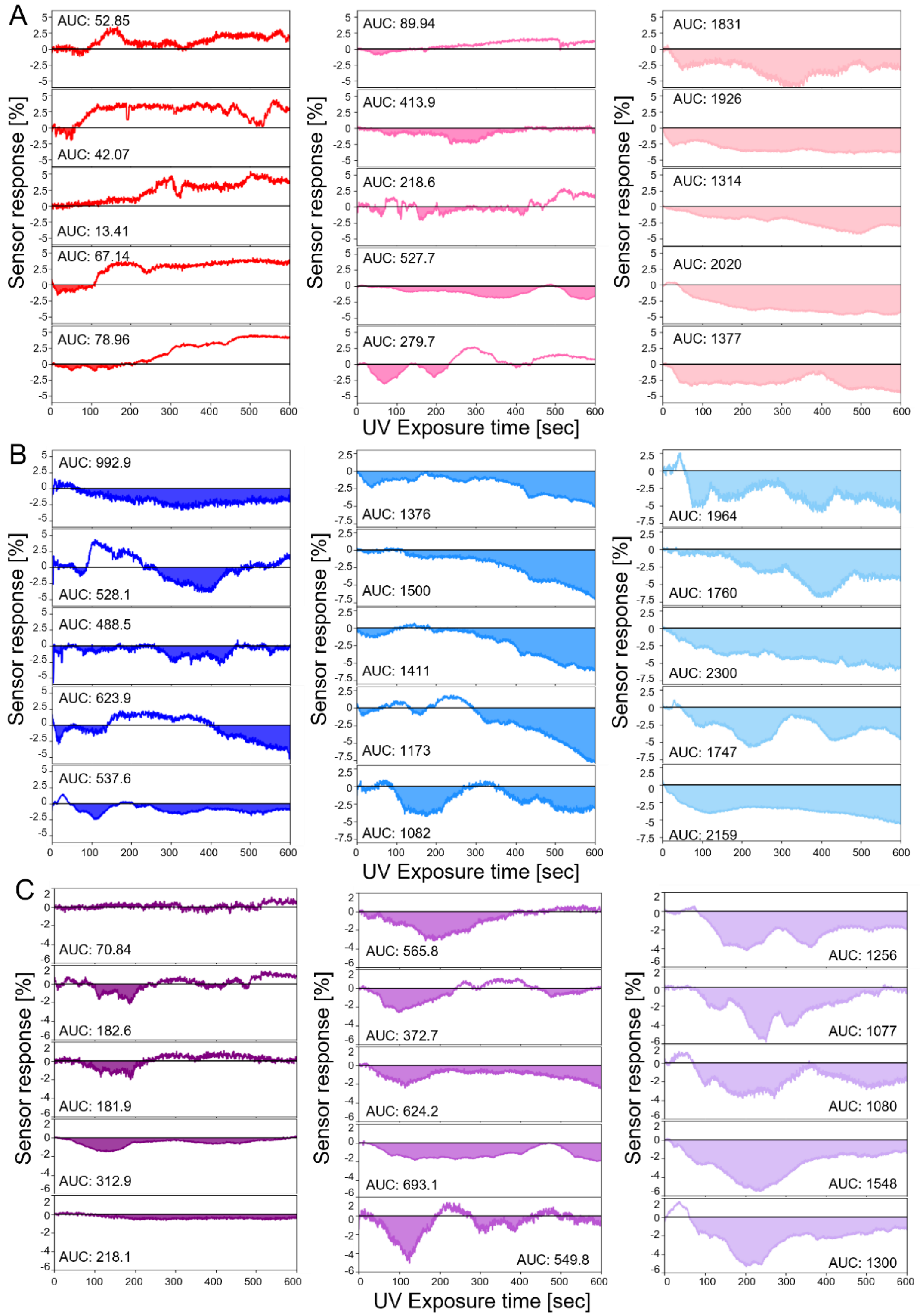

Fig. S32. Antioxidative working curve of vitamin C, vitamin E, and FBGE. Antioxidative time-series sensor response data and AUC value for each sample treated with (A) 50 μM, 5 μM and 0.5 μM vitamin C (left to right). (**B**) and (**C**) correspond to 100 μg/mL, 10 μg/mL, 1 μg/mL vitamin E, and 1,000 ppm, 100 ppm, 10 ppm FBGE, respectively (left to right).

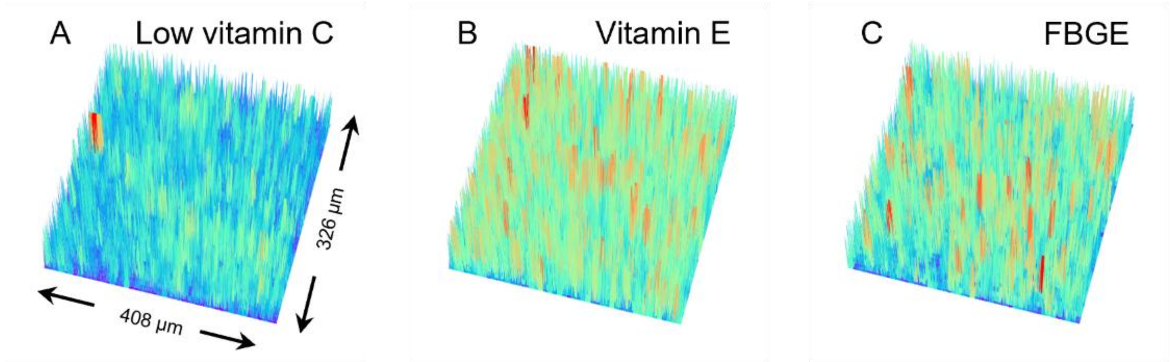

Fig. S33. 3D signal mapping of photoaging snapshot of pretreatment with cosmetic ingredients. (**A**) Vitamin C 5 μM. (**B**) Vitamin E 100 μg/mL. (**C**) FBFE 1,000 ppm.

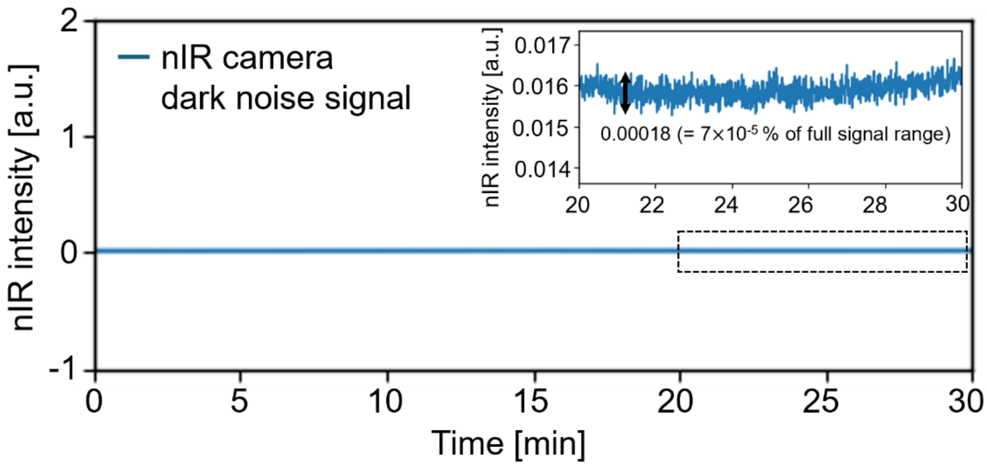

Fig. S34. Dark noise measurement of the nIR camera over a 30-minute acquisition. The noise amplitude was 0.00018, corresponding to approximately 7×10^{-5} % of the full signal range (0–255). The long-term baseline shift was measured to be 0.016, equivalent to 6×10^{-3} %, indicating stable signal acquisition without significant drift.

Ref	SWNT corona	Target molecules [Selectivity test]	H_2O_2 wave modeling	Spatial resolution	Sensing location	Application
37	Single [$(GT)_{15}$]	H_2O_2, $^1O_2$, ·OH [Not tested]	No	Single cell & Selected pixels	Extracellular & Intracellular	No /Biological mechanism study
44	Single [$(GT)_{15}$]	H_2O_2 [Not tested]	No	Single cell & Selected pixels	Extracellular	No /Cellular property measurement
43	Single [Collagen]	H_2O_2 [against H^+, NO_2^-, NO_3^-, $^1O_2$, O_2^- (NO – reverse rate comparison)]	No	Single cell & Selected pixels	Extracellular	No /Biological mechanism study
90	Single [Collagen]	H_2O_2 [Not tested]	No	Single cell & Selected pixels	Extracellular	No /Biological stress response study
91	Single [$(AT)_{15}$]	NO [Not tested]	No	Single cell & Selected pixels	Extracellular	No /Biological stress response study
**This work**	**Dual [PLL/$(CCCT)_7$]**	H_2O_2 **[against O_2^-, $^1O_2$, ·OH, NO, NO_2^-, NO_3^-, NH_4^+]**	**Yes [Log-normal probability density function]**	**From single cell to multiple cells & Whole pixels**	Extracellular	**Yes [Platform for evaluating cosmetic antioxidants]**

Table S1. Comparison between previous cellular H_2O_2 monitoring using SWNT sensor. The reference numbers are ordered according to the sequence in the manuscript.

Movie S1.
nIR Fluorescence of SNI under 6.6 J/cm^2 UVA (220 $mJ/cm^2 \cdot min$).

Movie S2.
nIR Fluorescence of SNI under 2.4 J/cm^2 UVA (80 $mJ/cm^2 \cdot min$).

Movie S3.
nIR Fluorescence of SNI with cells densely populated at the center.

Movie S4.
nIR Fluorescence of SNI with cells sparsely populated.

Movie S5.
Antioxidative effect of FBGE under 6.6 J/cm^2 UVA (220 $mJ/cm^2 \cdot min$).

REFERENCES AND NOTES

1. C. Battie, S. Jitsukawa, F. Bernerd, S. Del Bino, C. Marionnet, M. Verschoore, New insights in photoaging, UVA induced damage and skin types. *Exp. Dermatol.* **23**, 7–12 (2014).

2. J. Krutmann, Ultraviolet A radiation-induced biological effects in human skin: Relevance for photoaging and photodermatosis. *J. Dermatol. Sci.* **23**, S22–S26 (2000).

3. N. R. Attard, P. Karran, UVA photosensitization of thiopurines and skin cancer in organ transplant recipients. *Photochem. Photobiol. Sci.* **11**, 62–68 (2012).

4. J. Cadet, T. Douki, J.-L. Ravanat, P. Di Mascio, Sensitized formation of oxidatively generated damage to cellular DNA by UVA radiation. *Photochem. Photobiol. Sci.* **8**, 903–911 (2009).

5. M. S. Baptista, J. Cadet, A. Greer, A. H. Thomas, Photosensitization reactions of biomolecules: Definition, targets and mechanisms. *Photochem. Photobiol.* **97**, 1456–1483 (2021).

6. V. T. Natarajan, P. Ganju, A. Ramkumar, R. Grover, R. S. Gokhale, Multifaceted pathways protect human skin from UV radiation. *Nat. Chem. Biol.* **10**, 542–551 (2014).

7. T. Quan, Z. Qin, W. Xia, Y. Shao, J. J. Voorhees, G. J. Fisher, in *Journal of Investigative Dermatology Symposium Proceedings*. (Elsevier, 2009), vol. 14, pp. 20–24.

8. E. Steingrímsson, N. G. Copeland, N. A. Jenkins, Melanocytes and the microphthalmia transcription factor network. *Annu. Rev. Genet.* **38**, 365–411 (2004).

9. J. Le Digabel, S. Houriez-Gombaud-Saintonge, J. Filiol, C. Lauze, G. Josse, Dermal fiber structures and photoaging. *J. Biomed. Opt.* **23**, 1–12 (2018).

10. A. Weihermann, M. Lorencini, C. Brohem, C. De Carvalho, Elastin structure and its involvement in skin photoageing. *Int. J. Cosmet. Sci.* **39**, 241–247 (2017).

11. Y. Miyamura, S. G. Coelho, R. Wolber, S. A. Miller, K. Wakamatsu, B. Z. Zmudzka, S. Ito, C. Smuda, T. Passeron, W. Choi, Regulation of human skin pigmentation and responses to ultraviolet radiation. *Pigment Cell Res.* **20**, 2–13 (2007).

12. D. E. Brash, A. Ziegler, A. S. Jonason, J. A. Simon, S. Kunala, D. J. Leffell, in *The journal of investigative dermatology. Symposium proceedings*. (1996), vol. 1, pp. 136–142.

13. M. Hollstein, D. Sidransky, B. Vogelstein, C. C. Harris, p53 mutations in human cancers. *Science* **253**, 49–53 (1991).

14. D. E. Brash, J. A. Rudolph, J. A. Simon, A. Lin, G. J. McKenna, H. P. Baden, A. J. Halperin, J. Pontén, A role for sunlight in skin cancer: UV-induced p53 mutations in squamous cell carcinoma. *Proc. Natl. Acad. Sci. U.S.A.* **88**, 10124–10128 (1991).

15. A. H. Huang, A. L. Chien, Photoaging: A review of current literature. *Curr. Dermatol. Rep.* **9**, 22–29 (2020).

16. P. Peres, V. Terra, F. Guarnier, R. Cecchini, A. Cecchini, Photoaging and chronological aging profile: Understanding oxidation of the skin. *J. Photochem. Photobiol. B Biol.* **103**, 93–97 (2011).

17. A. Kammeyer, R. Luiten, Oxidation events and skin aging. *Ageing Res. Rev.* **21**, 16–29 (2015).

18. S. Sun, P. Jiang, W. Su, Y. Xiang, J. Li, L. Zeng, S. Yang, Wild chrysanthemum extract prevents UVB radiation-induced acute cell death and photoaging. *Cytotechnology* **68**, 229–240 (2016).

19. M. Deng, Y. Xu, Z. Yu, X. Wang, Y. Cai, H. Zheng, W. Li, W. Zhang, Protective effect of fat extract on UVB-induced photoaging *in vitro* and *in vivo*. *Oxid. Med. Cell. Longev.* **2019**, 6146942 (2019).

20. W. Klinngam, P. Rungkamoltip, R. Wongwanakul, J. Joothamongkhon, S. Du-a-man, M. Khongkow, U. Asawapirom, T. Iempridee, U. Ruktanonchai, Skin rejuvenation efficacy and safety evaluation of *Kaempferia parviflora* standardized extract (BG100) in human 3D skin models and clinical trial. *Biomolecules* **14**, 776 (2024).

21. Z. Xiao, S. Yang, J. Chen, C. Li, C. Zhou, P. Hong, S. Sun, Z.-J. Qian, Trehalose against UVB-induced skin photoaging by suppressing MMP expression and enhancing procollagen I synthesis in HaCaT cells. *J. Funct. Foods* **74**, 104198 (2020).

22. S. Seité, C. Medaisko, F. Christiaens, C. Bredoux, D. Compan, H. Zucchi, D. Lombard, A. Fourtanier, Biological effects of simulated ultraviolet daylight: A new approach to investigate daily photoprotection. *Photodermatol. Photoimmunol. Photomed.* **22**, 67–77 (2006).

23. A. P. Demchenko, Photobleaching of organic fluorophores: Quantitative characterization, mechanisms, protection. *Methods Appl. Fluoresc.* **8**, 022001 (2020).

24. B. Cui, Y. Wang, J. Jin, Z. Yang, R. Guo, X. Li, L. Yang, Z. Li, Resveratrol treats UVB-induced photoaging by anti-MMP expression, through anti-inflammatory, antioxidant, and antiapoptotic properties, and treats photoaging by upregulating VEGF-B expression. *Oxid. Med. Cell. Longev.* **2022**, 6037303 (2022).

25. C. Y. R. Tan, C. L. Tan, T. Chin, M. Morenc, C. Y. Ho, H. A. Rovito, L. S. Quek, A. L. Soon, J. S. Lim, O. Dreesen, Nicotinamide prevents UVB- and oxidative stress−induced photoaging in human primary keratinocytes. *J. Invest. Dermatol.* **142**, 1670–1681.e12 (2022).

26. S. Fan, L. Lopez Llorens, F. P. Perona Martinez, R. Schirhagl, Quantum sensing of free radical generation in mitochondria of human keratinocytes during UVB exposure. *ACS Sens.* **9**, 2440–2446 (2024).

27. H. Fu, Y. Zhang, Q. An, D. Wang, S. You, D. Zhao, J. Zhang, C. Wang, M. Li, Anti-photoaging effect of Rhodiola rosea fermented by *Lactobacillus plantarum* on UVA-damaged fibroblasts. *Nutrients* **14**, 2324 (2022).

28. M. Zhang, T. Zhang, Y. Tang, G. Ren, Y. Zhang, X. Ren, Concentrated growth factor inhibits UVA-induced photoaging in human dermal fibroblasts via the MAPK/AP-1 pathway. *Biosci. Rep.* **40**, BSR20193566 (2020).

29. C. Marionnet, C. Pierrard, F. Lejeune, J. Sok, M. Thomas, F. Bernerd, Different oxidative stress response in keratinocytes and fibroblasts of reconstructed skin exposed to non extreme daily-ultraviolet radiation. *PLOS ONE* **5**, e12059 (2010).

30. R. Jiang, X. Xu, Z. Sun, F. Wang, R. Ma, K. Feng, T. Li, L. Sun, Protective effects of ginseng proteins on photoaging of mouse fibroblasts induced by UVA. *Photochem. Photobiol.* **96**, 113–123 (2020).

31. J. Zhang, M. P. Landry, P. W. Barone, J.-H. Kim, S. Lin, Z. W. Ulissi, D. Lin, B. Mu, A. A. Boghossian, A. J. Hilmer, A. Rwei, A. C. Hinckley, S. Kruss, M. A. Shandell, N. Nair, S. Blake, F. Şen, S. Şen, R. G. Croy, D. Li, K. Yum, J. H. Ahn, H. Jin, D. A. Heller, J. M. Essigmann, D. Blankschtein, M. S. Strano, Molecular recognition using corona phase complexes made of synthetic polymers adsorbed on carbon nanotubes. *Nat. Nanotechnol.* **8**, 959–968 (2013).

32. Y. Lee, W. Kim, Y. Cho, M. Yoon, S. Lee, J. Lee, S. Oh, Y. Song, B. J. Lee, Y. Kim, S. Y. Cho, Rational design of 3D polymer corona interfaces of single-walled carbon nanotubes for receptor-free virus recognition. *ACS Nano* **18**, 13214–13225 (2024).

33. A. Khademhosseini, K. Y. Suh, J. M. Yang, G. Eng, J. Yeh, S. Levenberg, R. Langer, Layer-by-layer deposition of hyaluronic acid and poly-L-lysine for patterned cell co-cultures. *Biomaterials* **25**, 3583–3592 (2004).

34. O. Song, Y. Cho, S.-Y. Cho, J. Kang, Solution-processing approach of nanomaterials toward an artificial sensory system. *Int. J. Extrem. Manuf.* **6**, 052001 (2024).

35. S. M. Bachilo, M. S. Strano, C. Kittrell, R. H. Hauge, R. E. Smalley, R. B. Weisman, Structure-assigned optical spectra of single-walled carbon nanotubes. *Science* **298**, 2361–2366 (2002).

36. H. Liu, D. Nishide, T. Tanaka, H. Kataura, Large-scale single-chirality separation of single-wall carbon nanotubes by simple gel chromatography. *Nat. Commun.* **2**, 309 (2011).

37. D. A. Heller, H. Jin, B. M. Martinez, D. Patel, B. M. Miller, T.-K. Yeung, P. V. Jena, C. Höbartner, T. Ha, S. K. Silverman, M. S. Strano, Multimodal optical sensing and analyte specificity using single-walled carbon nanotubes. *Nat. Nanotechnol.* **4**, 114–120 (2009).

38. G. Dukovic, B. E. White, Z. Zhou, F. Wang, S. Jockusch, M. L. Steigerwald, T. F. Heinz, R. A. Friesner, N. J. Turro, L. E. Brus, Reversible surface oxidation and efficient luminescence

quenching in semiconductor single-wall carbon nanotubes. *J. Am. Chem. Soc.* **126**, 15269–15276 (2004).

39. C. Song, P. E. Pehrsson, W. Zhao, Recoverable solution reaction of HiPco carbon nanotubes with hydrogen peroxide. *J. Phys. Chem. B.* **109**, 21634–21639 (2005).

40. Y. Tanaka, K. Hirayama, Y. Niidome, N. Nakashima, Determination of electronic states of individually dissolved (*n*,*m*) single-walled carbon nanotubes in solution. *Chem. Phys. Lett.* **482**, 114–117 (2009).

41. C. Eggeling, J. Widengren, R. Rigler, C. A. Seidel, Photobleaching of fluorescent dyes under conditions used for single-molecule detection: Evidence of two-step photolysis. *Anal. Chem.* **70**, 2651–2659 (1998).

42. D. A. Heller, S. Baik, T. E. Eurell, M. S. Strano, Single-walled carbon nanotube spectroscopy in live cells: Towards long-term labels and optical sensors. *Adv. Mater.* **17**, 2793–2799 (2005).

43. H. Jin, D. A. Heller, M. Kalbacova, J.-H. Kim, J. Zhang, A. A. Boghossian, N. Maheshri, M. S. Strano, Detection of single-molecule H_2O_2 signalling from epidermal growth factor receptor using fluorescent single-walled carbon nanotubes. *Nat. Nanotechnol.* **5**, 302–309 (2010).

44. S.-Y. Cho, X. Gong, V. B. Koman, M. Kuehne, S. J. Moon, M. Son, T. T. S. Lew, P. Gordiichuk, X. Jin, H. D. Sikes, M. S. Strano, Cellular lensing and near infrared fluorescent nanosensor arrays to enable chemical efflux cytometry. *Nat. Commun.* **12**, 3079 (2021).

45. M. A. Lee, F. T. Nguyen, K. Scott, N. Y. L. Chan, N. A. Bakh, K. K. Jones, C. Pham, P. Garcia-Salinas, D. Garcia-Parraga, A. Fahlman, V. Marco, V. B. Koman, R. J. Oliver, L. W. Hopkins, C. Rubio, R. P. Wilson, M. G. Meekan, C. M. Duarte, M. S. Strano, Implanted nanosensors in marine organisms for physiological biologging: Design, feasibility, and species variability. *ACS Sens.* **4**, 32–43 (2018).

46. S. Kruss, M. P. Landry, E. Vander Ende, B. M. Lima, N. F. Reuel, J. Zhang, J. Nelson, B. Mu, A. Hilmer, M. Strano, Neurotransmitter detection using corona phase molecular recognition on fluorescent single-walled carbon nanotube sensors. *J. Am. Chem. Soc.* **136**, 713–724 (2014).

47. C. Marionnet, C. Tricaud, F. Bernerd, Exposure to non-extreme solar UV daylight: Spectral characterization, effects on skin and photoprotection. *Int. J. Mol. Sci.* **16**, 68–90 (2014).

48. D. E. Godar, UV Doses Worldwide. *Photochem. Photobiol.* **81**, 736–749 (2005).

49. J. B. Liley, R. L. McKenzie, Where on Earth has the highest UV. *UV Radiation and its Effects: An update* **68**, 36–37 (2006).

50. J. McGrath, R. Eady, F. Pope, Anatomy and organization of human skin. Rook's Textb. *Dermatology* **1**, 34–86 (2004).

51. T. T. S. Lew, V. B. Koman, K. S. Silmore, J. S. Seo, P. Gordiichuk, S.-Y. Kwak, M. Park, M. C.-Y. Ang, D. T. Khong, M. A. Lee, M. B. Chan-Park, N. H. Chua, M. S. Strano, Real-time detection of wound-induced H_2O_2 signalling waves in plants with optical nanosensors. *Nat Plants* **6**, 404–415 (2020).

52. A. Weidinger, A. V. Kozlov, Biological activities of reactive oxygen and nitrogen species: Oxidative stress versus signal transduction. *Biomolecules* **5**, 472–484 (2015).

53. K. Jomova, R. Raptova, S. Y. Alomar, S. H. Alwasel, E. Nepovimova, K. Kuca, M. Valko, Reactive oxygen species, toxicity, oxidative stress, and antioxidants: Chronic diseases and aging. *Arch. Toxicol.* **97**, 2499–2574 (2023).

54. H. J. Forman, M. Torres, Reactive oxygen species and cell signaling: Respiratory burst in macrophage signaling. *Am. J. Respir. Crit. Care Med.* **166**, S4–S8 (2002).

55. C. Espinosa-Diez, V. Miguel, D. Mennerich, T. Kietzmann, P. Sánchez-Pérez, S. Cadenas, S. Lamas, Antioxidant responses and cellular adjustments to oxidative stress. *Redox Biol.* **6**, 183–197 (2015).

56. H. Sies, Hydrogen peroxide as a central redox signaling molecule in physiological oxidative stress: Oxidative eustress. *Redox Biol.* **11**, 613–619 (2017).

57. J. A. Imlay, Cellular defenses against superoxide and hydrogen peroxide. *Annu. Rev. Biochem.* **77**, 755–776 (2008).

58. J. Kumamoto, S. Nakanishi, M. Makita, M. Uesaka, Y. Yasugahira, Y. Kobayashi, M. Nagayama, S. Denda, M. Denda, Mathematical-model-guided development of full-thickness epidermal equivalent. *Sci. Rep.* **8**, 17999 (2018).

59. S. Kruss, D. P. Salem, L. Vuković, B. Lima, E. Vander Ende, E. S. Boyden, M. S. Strano, High-resolution imaging of cellular dopamine efflux using a fluorescent nanosensor array. *Proc. Natl. Acad. Sci. U.S.A.* **114**, 1789–1794 (2017).

60. J. Ackermann, E. Reger, S. Jung, J. Mohr, S. Herbertz, K. Seidl, S. Kruss, Smart slides for optical monitoring of cellular processes. *Adv. Funct. Mater.* **34**, 2309064 (2024).

61. R. Nißler, A. T. Müller, F. Dohrman, L. Kurth, H. Li, E. G. Cosio, B. S. Flavel, J. P. Giraldo, A. Mithöfer, S. Kruss, Detection and imaging of the plant pathogen response by near-infrared fluorescent polyphenol sensors. *Angew. Chem. Int. Ed. Engl.* **61**, e202108373 (2022).

62. S. I. Zandalinas, R. Mittler, ROS-induced ROS release in plant and animal cells. *Free Radic. Biol. Med.* **122**, 21–27 (2018).

63. N. Dutta, G. Garcia, R. Higuchi-Sanabria, Hijacking cellular stress responses to promote lifespan. *Front. Aging* **3**, 860404 (2022).

64. S. Vanderauwera, N. Suzuki, G. Miller, B. van de Cotte, S. Morsa, J.-L. Ravanat, A. Hegie, C. Triantaphylidès, V. Shulaev, M. C. E. Van Montagu, F. Van Breusegem, R. Mittler, Extranuclear protection of chromosomal DNA from oxidative stress. *Proc. Natl. Acad. Sci. U.S.A.* **108**, 1711–1716 (2011).

65. A. M. Wolf, K. Nishimaki, N. Kamimura, S. Ohta, Real-time monitoring of oxidative stress in live mouse skin. *J. Invest. Dermatol.* **134**, 1701–1709 (2014).

66. W. Wang, H. Fang, L. Groom, A. Cheng, W. Zhang, J. Liu, X. Wang, K. Li, P. Han, M. Zheng, J. Yin, W. Wang, M. P. Mattson, J. P. Y. Kao, E. G. Lakatta, S.-S. Sheu, K. Ouyang, J. Chen, R. T. Dirksen, H. Cheng, Superoxide flashes in single mitochondria. *Cell* **134**, 279–290 (2008).

67. W. Wang, G. Gong, X. Wang, L. Wei-LaPierre, H. Cheng, R. Dirksen, S.-S. Sheu, Mitochondrial flash: Integrative reactive oxygen species and pH signals in cell and organelle biology. *Antioxid. Redox Signal.* **25**, 534–549 (2016).

68. H. Fang, M. Chen, Y. Ding, W. Shang, J. Xu, X. Zhang, W. Zhang, K. Li, Y. Xiao, F. Gao, S. Shang, J. C. Li, X. L. Tian, S. Q. Wang, J. Zhou, N. Weisleder, J. Ma, K. Ouyang, J. Chen, X. Wang, M. Zheng, W. Wang, X. Zhang, H. Cheng, Imaging superoxide flash and metabolism-coupled mitochondrial permeability transition in living animals. *Cell Res.* **21**, 1295–1304 (2011).

69. A. V. Kuznetsov, S. Javadov, V. Saks, R. Margreiter, M. Grimm, Synchronism in mitochondrial ROS flashes, membrane depolarization and calcium sparks in human carcinoma cells. *Biochim. Biophys. Acta Bioenerg.* **1858**, 418–431 (2017).

70. E. W. Miller, B. C. Dickinson, C. J. Chang, Aquaporin-3 mediates hydrogen peroxide uptake to regulate downstream intracellular signaling. *Proc. Natl. Acad. Sci. U.S.A.* **107**, 15681–15686 (2010).

71. G. Nelson, J. Wordsworth, C. Wang, D. Jurk, C. Lawless, C. Martin-Ruiz, T. von Zglinicki, A senescent cell bystander effect: Senescence-induced senescence. *Aging Cell* **11**, 345–349 (2012).

72. R. Mittler, S. I. Zandalinas, Y. Fichman, F. Van Breusegem, Reactive oxygen species signalling in plant stress responses. *Nat. Rev. Mol. Cell Biol.* **23**, 663–679 (2022).

73. M. Garmyn, A. Young, S. Miller, Mechanisms of and variables affecting UVR photoadaptation in human skin. *Photochem. Photobiol. Sci.* **17**, 1932–1940 (2018).

74. J.-C. Hervé, M. Derangeon, Gap-junction-mediated cell-to-cell communication. *Cell Tissue Res.* **352**, 21–31 (2013).

75. L. Galluzzi, T. Yamazaki, G. Kroemer, Linking cellular stress responses to systemic homeostasis. *Nat. Rev. Mol. Cell Biol.* **19**, 731–745 (2018).

76. Y. Lin, Z. Cao, T. Lyu, T. Kong, Q. Zhang, K. Wu, Y. Wang, J. Zheng, Single-cell RNA-seq of UVB-radiated skin reveals landscape of photoaging-related inflammation and protection by vitamin D. *Gene* **831**, 146563 (2022).

77. S. Oh, S. Zheng, M. Fang, M. Kim, A. D. Bellere, J. Jeong, T.-H. Yi, Anti-photoaging effect of *Phaseolus angularis* L. extract on UVB-exposed HaCaT keratinocytes and possibilities as cosmetic materials. *Molecules* **28**, 1407 (2023).

78. Y. Kim, J. Sim, K. Jeon, D. Ryu, Y. Ji, Y. Kim, J. Kim, S. Jeon, D. Park, E. Jung, Fermented black ginseng extract prevents UVB-induced inflammation by regulating the nc886-PKR pathway in human keratinocytes. *Photodermatol. Photoimmunol. Photomed.* **40**, 10.1111/phpp.12927 (2024).

79. M. Levine, Y. Wang, S. J. Padayatty, J. Morrow, A new recommended dietary allowance of vitamin C for healthy young women. *Proc. Natl. Acad. Sci.* **98**, 9842–9846 (2001).

80. M. G. Traber, Vitamin E regulatory mechanisms. *Annu. Rev. Nutr.* **27**, 347–362 (2007).

81. M. V. Catani, I. Savini, A. Rossi, G. Melino, L. Avigliano, Biological role of vitamin C in keratinocytes. *Nutr. Rev.* **63**, 81–90 (2005).

82. S. Maalouf, M. El-Sabban, N. Darwiche, H. Gali-Muhtasib, Protective effect of vitamin E on ultraviolet B light-induced damage in keratinocytes. *Mol. Carcinog.* **34**, 121–130 (2002).

83. A. J. Didier, J. Stiene, L. Fang, D. Watkins, L. D. Dworkin, J. F. Creeden, Antioxidant and anti-tumor effects of dietary vitamins A, C, and E. *Antioxidants* **12**, 632 (2023).

84. Y. Tian, X. Zhang, M. Du, F. Li, M. Xiao, W. Zhang, Synergistic antioxidant effects of araloside A and L-ascorbic ccid on H_2O_2-induced HEK293 cells: Regulation of cellular antioxidant status. *Oxid. Med. Cell. Longev.* **2021**, 9996040 (2021).

85. A. Gazel, P. Ramphal, M. Rosdy, C. Tornier, N. Hosein, B. Lee, M. Tomic-Canic, M. Blumenberg, Transcriptional profiling of epidermal keratinocytes: Comparison of genes expressed in skin, cultured keratinocytes, and reconstituted epidermis, using large DNA microarrays. *J. Invest. Dermatol.* **121**, 1459–1468 (2003).

86. J. P. Smits, H. Niehues, G. Rikken, I. M. van Vlijmen-Willems, G. W. van de Zande, P. L. Zeeuwen, J. Schalkwijk, E. H. van den Bogaard, Immortalized N/TERT keratinocytes as an alternative cell source in 3D human epidermal models. *Sci. Rep.* **7**, 11838 (2017).

87. N. Barua, L. Huang, C. Li, Y. Yang, M. Luo, W. I. Wei, K. T. Wong, N. W. S. Lo, K. O. Kwok, M. Ip, Comparative study of two-dimensional (2D) vs. three-dimensional (3D) organotypic kertatinocyte-fibroblast skin models for *Staphylococcus aureus* (MRSA) infection. *Int. J. Mol. Sci.* **23**, 299 (2021).

88. D. S. Rosenthal, L.-W. Kuo, S. L. Seagrave, V. Soni, N. Islam, G. Minsky, L. Dussan-Cuellar, B. Ell, C. M. Simbulan-Rosenthal, P. Sykora, Skin immuno-CometChip in 3D vs. 2D cultures to screen topical toxins and skin-specific cytochrome inducers. *Genes* **14**, 630 (2023).

89. J. Liu, W. Zhang, The influence of the environment and clothing on human exposure to ultraviolet light. *PLOS ONE* **10**, e0124758 (2015).

90. J.-H. Kim, C. R. Patra, J. R. Arkalgud, A. A. Boghossian, J. Zhang, J.-H. Han, N. F. Reuel, J.-H. Ahn, D. Mukhopadhyay, M. S. Strano, Single-molecule detection of H_2O_2 mediating angiogenic redox signaling on fluorescent single-walled carbon nanotube array. *ACS Nano* **5**, 7848–7857 (2011).

91. Z. W. Ulissi, F. Sen, X. Gong, S. Sen, N. Iverson, A. A. Boghossian, L. C. Godoy, G. N. Wogan, D. Mukhopadhyay, M. S. Strano, Spatiotemporal intracellular nitric oxide signaling captured using internalized, near-infrared fluorescent carbon nanotube nanosensors. *Nano Lett.* **14**, 4887–4894 (2014).
